# Unlocking Brightness in CsPbCl_3_ Perovskite Nanocrystals: Screening Ligands and Metal Halides for Effective Deep Trap Passivation

**DOI:** 10.1021/acsenergylett.5c00185

**Published:** 2025-03-12

**Authors:** Nadesh Fiuza-Maneiro, Junzhi Ye, Shilendra Kumar Sharma, Sudip Chakraborty, Sergio Gómez-Graña, Robert L. Z. Hoye, Lakshminarayana Polavarapu

**Affiliations:** † 130363 CINBIO, Universidade de Vigo, Materials Chemistry and Physics Group, Department of Physical Chemistry, Campus Universitario Lagoas-Marcosende, 36310 Vigo, Spain; ‡ Inorganic Chemistry Laboratory, 6396 University of Oxford, South Parks Road, Oxford OX1 3QR, United Kingdom; # Materials Theory for Energy Scavenging (MATES) Lab, Department of Physics, 56957 Harish-Chandra Research Institute (HRI) Allahabad, A C.I. of Homi Bhabha National Institute (HBNI), Chhatnag Road Jhunsi, Prayagraj 211019, India

## Abstract

Despite the significant advances made in achieving green (CsPbBr_3_)- and red (CsPbI_3_)-emitting halide perovskite nanocrystals (NCs) with high quantum yields and colloidal stability through surface engineering, obtaining bright violet/blue-emitting CsPbCl_3_ NCs with long-term stability is still a grand challenge due to their defect sensitivity. In this work, we have screened the surface passivation of CsPbCl_3_ NCs using ligands with different functional groups (amine, sulfonic, and phosphonic acid) and metal halides (mono- and bivalent) with the aim of improving the emission yield and stability of CsPbCl_3_ NCs. This enabled us to find that phosphonic acids are the ligands that showed the highest efficiency as they occupy Cl vacancies and covalently bind to the Pb on the surface of NCs, together with the incorporation of bivalent metal chlorides that showed substantial enhancements in PLQY. Consequently, the most effective passivators were those that passivate Cl vacancies, indicating these to be among the most detrimental traps. This is further validated through Density Functional Theory (DFT), suggesting that the trend in adsorption energies is as follows: hexylphosphonic < hexylsulfonic < oleylamine < tetrabutyl ammonium, which is also coherent with the charge transfer mechanism and corresponding electronic structure of the halide perovskite surface with the ligands. Furthermore, after evaluating different passivation strategies, we identified *in situ* passivation as the most effective method for obtaining highly luminescent CsPbCl_3_ NCs that exhibit stability for over 6 months. Thus, this work is expected to guide the perovskite NC researchers to choose effective passivating agents and passivation strategies toward bright blue luminescent colloidal halide perovskites and beyond.

## Graphical abstract

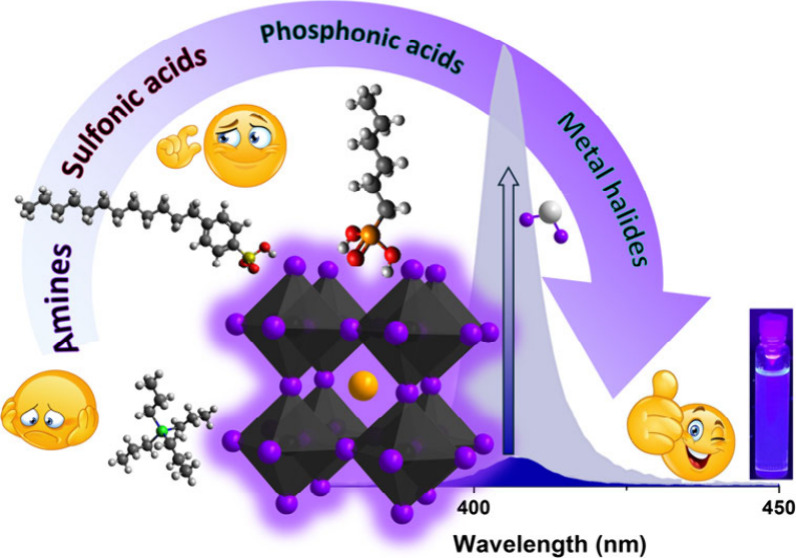

Lead halide perovskite nanocrystals (NCs) have recently gained much interest due to their excellent optical and optoelectronic properties, making them suitable for applications in LEDs, photovoltaic cells and other optoelectronic devices.^1–5^ One of the key features of these materials compared to traditional semiconductors is their exceptional defect tolerance for both band-edge and hot carriers.^6^ This is primarily attributed to the formation of shallow traps with low capture cross sections.^4^ As a result, these materials exhibit significantly reduced trap-assisted recombination, even in the presence of a high defect density. Some of the factors contributing to this are the high dielectric constants due to the high Born effective charge of lead (dielectric screening),^7^ as well as, high spin–orbit coupling (due to the presence of heavy elements) that reduces the bandgap and makes it more likely that trap states will be shallow.^4,8,9^ The electronic structure at the band edges also favors shallow rather than deep trap formation.^4^ Nevertheless, defect tolerance is dependent not only on electronic structure but also on the crystal structure. For instance, a reduction in bond angles decreases the overlap between the Pb and I orbitals, leading to reduced defect tolerance in certain crystalline phases.^10,11^ Another structural factor to consider is the cation–anion bond length, which makes defect tolerance dependent on the halide composition. As the size of the halide decreases from iodide to chloride, the lattice parameter also decreases, resulting in a shorter lead-halide bond. In addition, the increased ionicity of this bond leads to a wider bandgap with a lower electron affinity, such that deep traps are more likely to form compared to their iodide-based counterparts.^11–13^ This is why the typical oleylammonium (OAm) -capped CsPbCl_3_ NCs exhibit PLQYs less than 1%, whereas CsPbBr_3_ and CsPbI_3_ NCs can exhibit ∼100% PLQY.

In addition to these intrinsic structural and chemical factors, external conditions such as aging, washing, and dilution can have further adverse effects on the luminescence efficiency of nanocrystals (NCs), as well as their stability. Consequently, numerous studies have emerged both by employing post-synthetic or *in situ* surface passivation strategies using ligands with high binding affinity or excess metal halides to fill these traps and enhance the optoelectronic properties of perovskite NCs.^14^ For bromide or iodide-based perovskite NCs, different ligands such as phosphorus based,^15–20^ sulfur based^21–31^ or amines,^32–41^ and metal salts^42–45^ have shown promising results for surface passivation. However, these remain relatively unexplored for CsPbCl_3_ NCs. Although there are few reports on the passivation of deep blue/violet-emitting CsPbCl_3_ NCs with ligands and metal salts,^46–48^ the rationale behind the use of passivators and the role of functional groups of ligands has not been well-established in the literature. This is because different passivators have been reported under different experimental conditions in different literature.^14^ Therefore, it is still unclear which ligands or metal halides (also, ligands vs metal halides) can effectively passivate the surface of perovskite NCs. In addition, which passivation method, *i.e*., *in situ* vs postsynthetic, is effective for obtaining bright perovskite NCs is still to be established. Besides, the long-term stability of bright CsPbCl_3_ is one of the outstanding challenges. To address these outstanding questions, we propose in this paper a comprehensive study of screening different ligands and metal halides to obtain bright and relatively stable CsPbCl_3_ NCs through *in situ* and postsynthetic surface passivation.

Our approach involves screening ligands with different functional groups, such as quaternary amines, phosphor or sulfur-based, carboxylic acids, and metal halide salts for surface passivation of CsPbCl_3_ NCs (Figure 1). These passivators have been very effective in obtaining CsPbBr_3_ NCs with high PLQYs and stability. We employed two different passivation strategies. First, we performed postsynthetic passivation in the screening of ligands and metal halides for effective passivation. Then, the most efficient passivating agents were selected for *in situ* passivation, allowing us to compare both methodologies. Through this dual approach, we aim to not only unveil the most effective passivation method but also establish a relationship between functional groups of the ligands/metal halides vs surface passivation.

**Figure 1:**
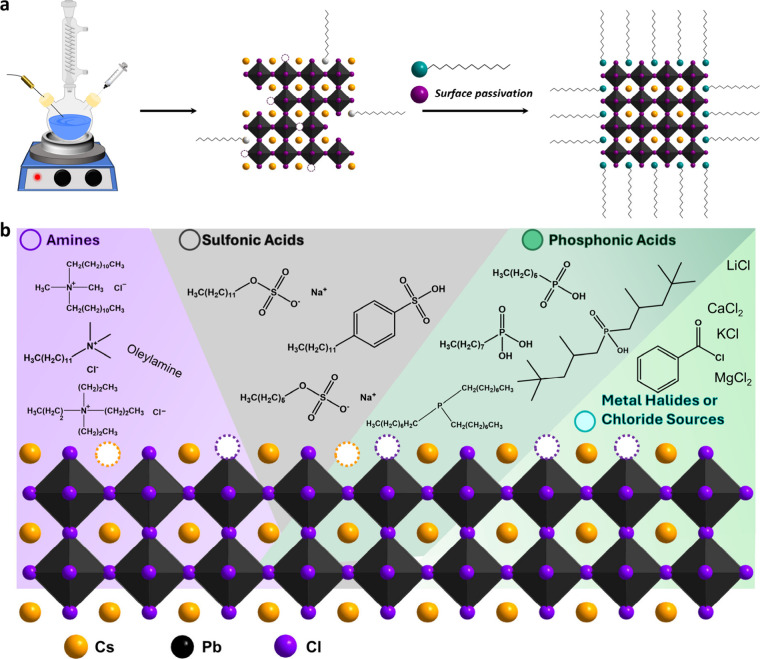
(a) Schematic of the post-synthetic surface passivation approach. (b) Illustration of the different ligands investigated for post-synthetic surface passivation. Amine-based ligands are highlighted in purple, sulfonic acids in gray, phosphonic acids in green, and metal halides or other chloride sources in blue.

## Results and Discussion

The synthesis of the CsPbCl_3_ NCs was carried out by the typical hot injection method at 180 °C, obtaining monodisperse NCs with an average size distribution of 6.08 ± 0.03 nm. The PL spectra exhibit a peak at 401 nm with a PLQY≈ of 1% (see Figure S1 for more information about the PLQY measurement protocol) with a full-width half-maximum (fwhm) of 17 nm (Figure S2, Supporting Information). We subsequently employed a range of passivating ligands with varying chain lengths, degrees of steric hindrance, and different metal halide salts (Figure 1b) to implement a post-synthetic surface passivation strategy (Figure 1a) to test their effectiveness according to their structure. Depending on their solubilities, we utilized two distinct approaches for passivation. For ligands, we prepared solutions of 2 mM in toluene, and a predetermined volume of the ligand solution was added to the pristine NCs at a ratio of 1.66:1, which was kept sonicating for 1 min (see scheme in Figure 2a). After sonication, ethyl acetate (EtOAc) antisolvent was added to precipitate the NCs and then proceeded to centrifugation for 10 min at 6,000 rpm to remove excess ligands. The precipitated NCs were then redispersed in toluene for further characterization (see Figures 2a–d and S3 for electron microscopy images of the samples). It is worth noting that in most reports, the NCs were not washed with an antisolvent after passivation.^49,50^ However, the washing step is critical to obtaining NC films for optoelectronic devices; otherwise, the excess ligands make the film sticky as well as prevent charge transport.

**Figure 2:**
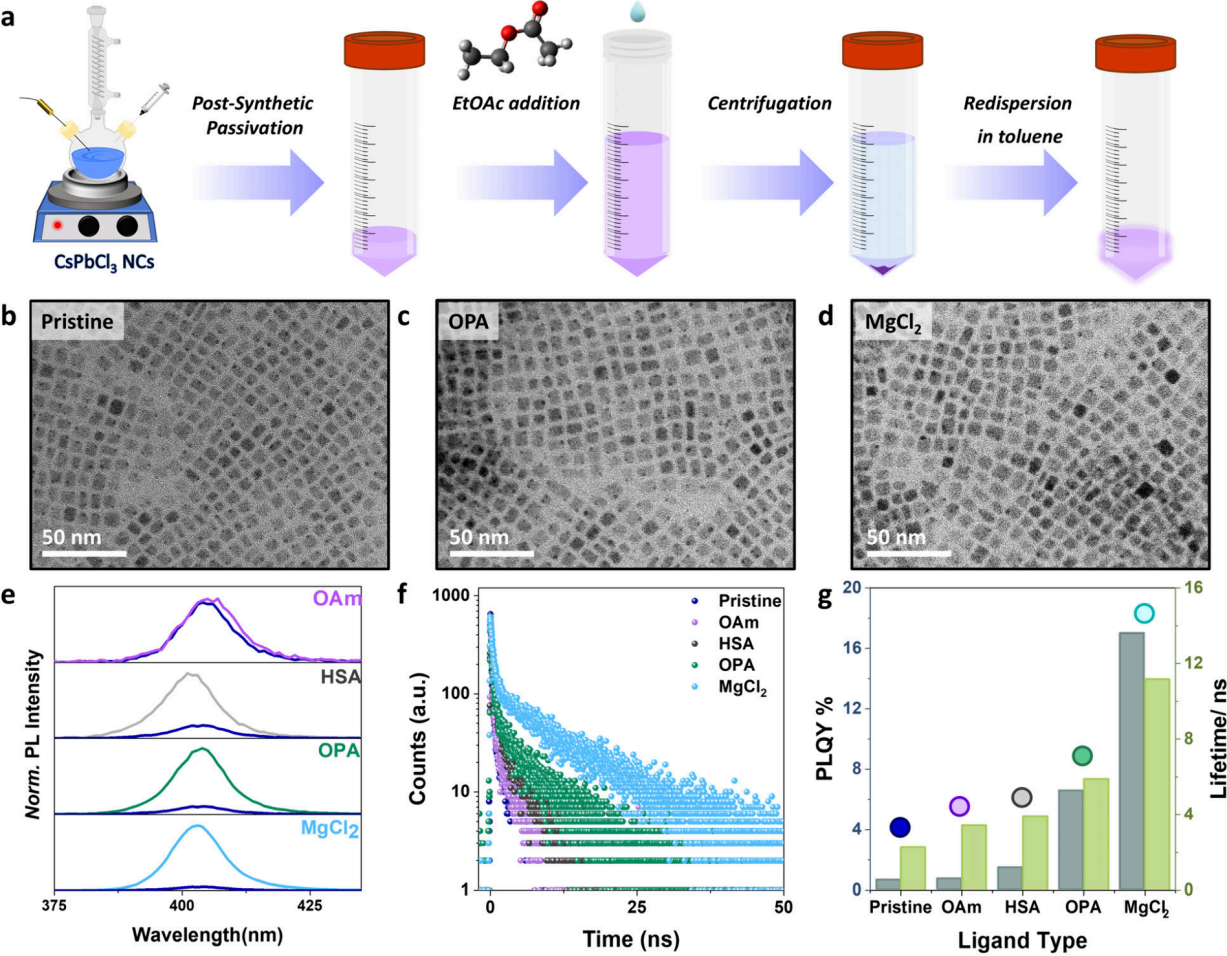
(a) Schematic illustration of the post-synthetic surface passivation approach used in this work. (b–d) TEM images of (b) pristine, (c) OPA- and (d) MgCl_2_-passivated samples. (e) PL spectra normalized relative to the absorption at the excitation wavelength of pristine (blue) NCs compared to NCs with different passivation treatments. (f) Time-resolved PL Lifetimes of pristine NCs, oleylamine-, sodium hexyl sulfonate-, octylphosphonic acid- and MgCl_2_-treated NCs. The excitation source is a nanosecond pulsed laser with a 350 nm wavelength (41.88 μJ/cm^2^ fluence). (g) PLQY and PL lifetimes of pristine and the passivated (post-synthetic) CsPbCl_3_ NCs.

After post-synthetic passivation, we characterized the samples to compare their efficiency in improving PLQY. The results showed a remarkable dependence on the functional groups of the ligands (Figure 2e–g, S4 (absorption and PL) and S5 (PL lifetimes)). The primary and quaternary amine ligands exhibited minimal changes in PL, and in some cases, even a decrease, suggesting that amines are not effective passivating ligands for CsPbCl_3_ NCs. This can be explained by the fact that amines tend to fill the Cs^+^ vacancies, which do not contribute to the electronic structure in the band-edge states and thus have little effect on the luminescence.^51^ In contrast, passivation with sulfonic acids results in a slight increase in the PLQY, with the highest improvement of over ×3.1 fold observed for sodium hexyl sulfonate (HSA), without showing a notable difference for longer chain sulfonate ligands (see Table S1 for more PLQY enhancement values). In addition, HSA passivation leads to a considerable increase in the PL lifetime, increasing from 2.27 ns for pristine NCs to 3.90 ns for passivated NCs (measured at 41.88 μJ/cm^2^fluence). In addition, it was considered interesting to examine the possibility that the sodium atoms of the sulfonate ligand might contribute to the passivation. However, XPS data show that the corresponding Na 1s peaks of free ligands and the passivated NCs overlap, suggesting that the Na ions are likely not incorporated into the NC lattice (Figure S6). In contrast, phosphorus-based ligands have yielded the most promising results, especially phosphonic acids with octyl phosphonic acid (OPA) at the forefront. After passivation with OPA, we achieved an enhancement factor of approximately 10, along with an increase of the lifetime to 5.9 ns, while preserving the morphology and crystalline structure, as confirmed by TEM images (Figure 2b, c, and more STEM images can be found in Figure S3). Similarly, other longer-chain phosphonic acids, like dodecyl phosphonic acid, also showed high enhancement factors of PLQY (≈ ×9), indicating that the chain length is not a critical factor for passivation and mainly depends on the functional group. Moreover, a ×2.5-fold increase in PLQY was observed when employing the phosphinic acid ligand Bis­(2,4,4-trimethylpentyl) phosphinic acid. The observed decrease in effectiveness may be attributed to the steric hindrance imposed by the molecular structure as well as the absence of free electron pairs. On the other hand, the phosphor-based ligands trioctylphosphine (TOP) and trioctylphosphine oxide (TOPO) showed no improvement of the emission properties.

This is consistent with previous results, suggesting that TOP/TOPO does not coordinate with NCs surface because it served as a coordination solvent to dissolve the lead halide.^22^ That passivation with sulfonic acid and phosphor-based compounds is more effective than using amines suggests that chloride vacancies are more detrimental to the optoelectronic properties, as these ligands are characterized by coordinating to negatively charged vacancies, whereas amines are more likely to passivate defects in cesium vacancies. Interestingly, a decrease in the absorption intensity was observed in proportion to the effectiveness of passivation (Figure S4), suggesting that the competitive ligand binding during the passivation process destabilized a fraction of the NCs. A slight bathochromic shift is observed in the OPA-passivated sample (Figure S4b), likely due to changes in polarity or electron density resulting from the donor–acceptor interactions between the phosphonic acid functional group and the Pb^2+^ centers.

Subsequently, we shifted our focus to screening different halide sources, starting with benzoyl chloride, which had already been used previously in a hot injection method with promising results.^52^ As expected, we observed a substantial enhancement with a factor of approximately 7. In the case of metal halide passivation, we employed an alternative approach inspired from the literature,^46^ by adding the solid reagent directly to the colloidal solution to passivate CsPbCl_3_ NCs. This was followed by high-speed centrifugation (14,000 rpm, 30 s) to precipitate the remaining salts that had not reacted with the NCs. We tested various monovalent metal halides such as CsCl, KCl and LiCl as well as divalent metal halides such as CaCl_2_ and MgCl_2_. After all samples were characterized, divalent metal halides exhibited better results. Notably, the improvements in terms of PLQY (17%) and PL lifetime (11.2 ns) for MgCl_2_-treated sample far exceeded those obtained by using the organic ligands. These results suggest that the most detrimental traps for the PLQY are those originating from chloride vacancies. It is important to highlight that, despite slight variations in PLQY of samples from batch to batch by employing the different passivators, the trend remains the same as shown in Table S1 (see the average PLQY obtained from different experiments).

After identifying the passivating agents that showed the best results, we proceeded to carry out an *in situ* passivation strategy adding the ligands during the NCs synthesis by the hot injection method, as schematically shown in Figure 3a. Similar to the results obtained with post-synthetic passivation, the incorporation of MgCl_2_ salt yielded the most significant enhancement of PL and lifetime of CsPbCl_3_ NCs (Figure 3b, c, and see Section S6 and Figure S5 for excitation fluence-dependent lifetime comparison of some passivator agents). X-ray diffraction (XRD) measurements confirmed that the crystal structure was preserved for both the *in situ* passivated samples (Figure 3d). To study their surface stability, we performed washing experiments, treating the samples with EtOAc antisolvent, followed by centrifugation. After each wash, PL measurements revealed a sharp decrease in the optical properties for the pristine sample and the one passivated with OPA, while the PL of the MgCl_2_ passivated sample is less affected by washing (Figure 3e), suggesting that the ligands are more sensitive to antisolvents and are easier to get detach as compared to lattice incorporated Mg and Cl atoms.

**Figure 3:**
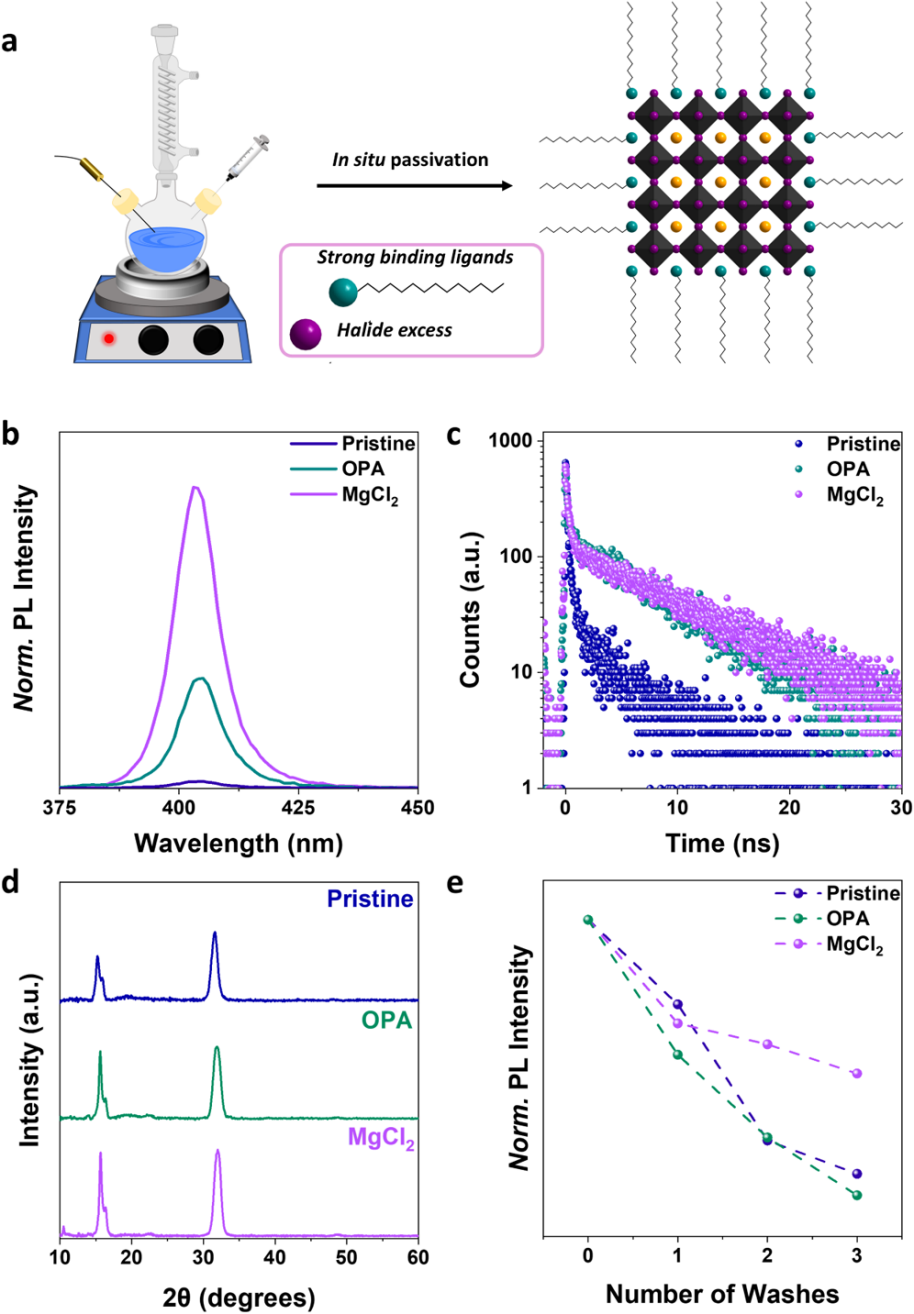
(a) Scheme of *in situ* passivation approach. (b) Normalized PL spectra relative to the absorption at the excitation wavelength of pristine and the *in situ* passivation with OPA and MgCl_2_. (c) Time-resolved PL Lifetimes for pristine, OPA, and MgCl_2_ treated samples. The excitation source is a nanosecond pulsed laser with a 350 nm wavelength (41.88 μJ/cm^2^ fluence). All samples were in colloidal solution, measured in a cuvette with a 1 mm-thick cavity. (d) XRD patterns for pristine, OPA, and MgCl_2_ *in situ* passivation samples. (e) Normalized PL spectra relative to the absorption at the excitation wavelength after washing with EtOAc as an antisolvent.

Furthermore, we carried out a more in-depth study of the synthesis (postsynthetic and *in situ* passivation) using the best passivators (OPA and MgCl_2_). First, focusing on the OPA ligand, we found that the *in situ* passivation significantly improved the PLQY and lifetime compared to the postsynthetic passivation, increasing the PLQY from 7 to 10% and the lifetime from 5.9 to 9.8 ns (Figure 4a). Scanning transmission electron microscopy (STEM) images confirmed the monodispersity of the NCs, with an average size of 6.8 ± 0.17 nm (Figure 4b). To confirm the binding of OPA to the surface of the NCs, we carried out ^31^P NMR experiments comparing the obtained CsPbCl_3_–OPA treated NCs with the free ligand. As shown in Figure 4c, an upfield shift could be observed from 38.41 to 23.13 ppm indicating the effective coordination of phosphonic acid to the lead, and filling the Cl vacancies (as it is shown in the scheme of Figure 4d) in agreement with previous literature.^15,53,54^ As observed in the magnified region, the peak of the OPA-treated sample exhibits broadening compared to that of the ligand, indicating its coordination to the nanocrystal surface. Furthermore, Fourier transform infrared spectroscopy (FTIR) data show a faint peak at around 1000 cm^–1^, which could be attributed to P–O stretching vibrations or P–OH bending characteristics of phosphonic acids (Figure S8). In addition, X-ray photoelectron spectroscopy (XPS) measurements confirmed the coordination of the phosphonic acid to the surface by showing a shift in the binding energy from 133.8 to 132.5 eV, indicating the presence of a new chemical environment relative to the free ligand (Figure S6c).

**Figure 4:**
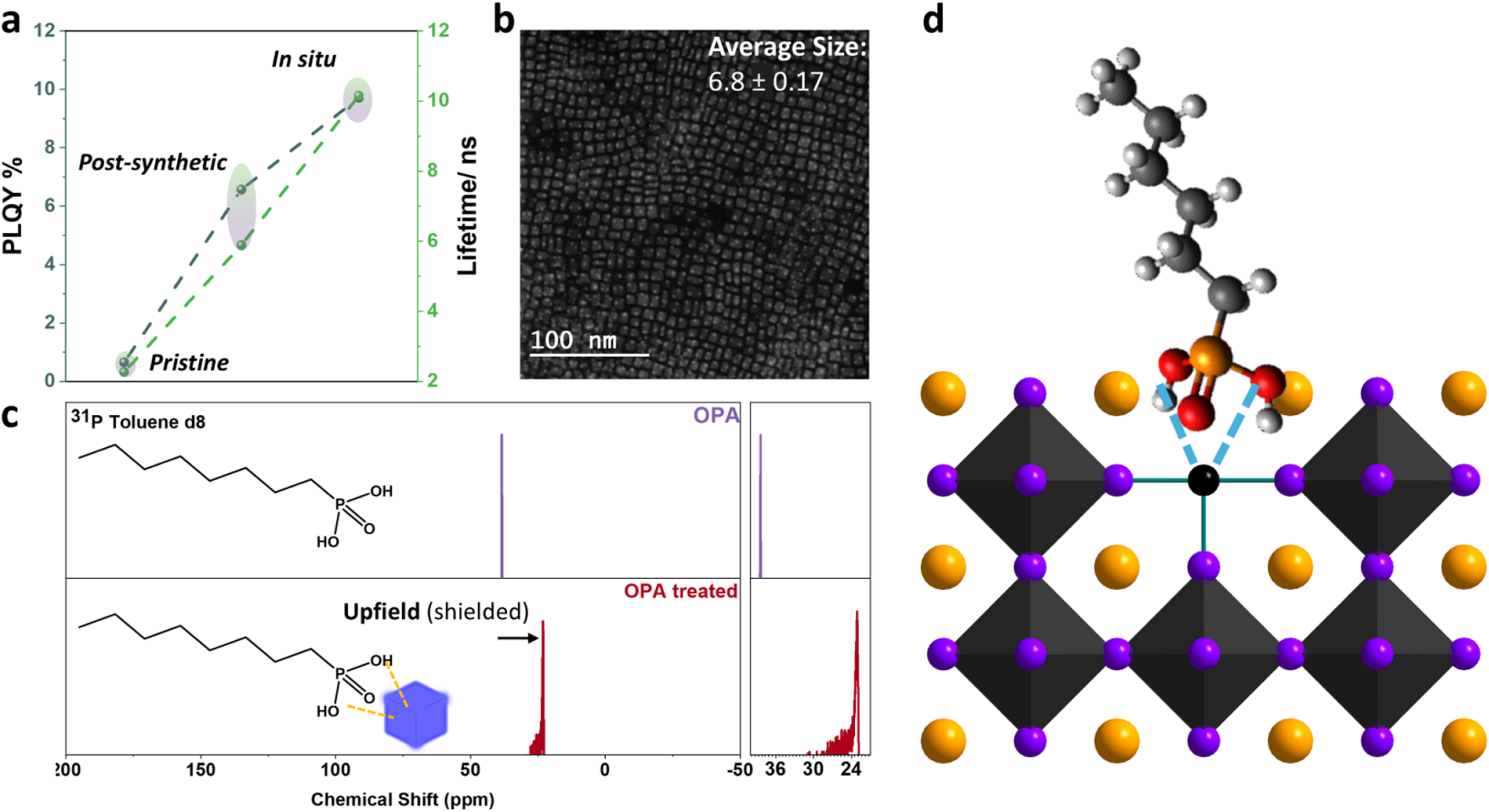
(a) A plot of PLQY vs PL lifetime for pristine and OPA-treated samples through in situ and post-synthetic passivation approaches. (b) STEM image of OPA *in situ* treated CsPbCl_3_ NCs. (c) ^31^P NMR experiments of free OPA ligand and CsPbCl_3_–OPA NCs. (d) Scheme of the dual surface passivation mechanism proposed for the OPA ligands.

We performed a similar experiment incorporating MgCl_2_ in a typical hot injection synthesis method. Once again, *in situ* passivation outperformed post-synthetic passivation, achieving a remarkable PLQY of 20.56% (enhancement factor of ×29) and a PL lifetime of 11.12 ns (Figure 5b). The incorporation of magnesium into the NC structure was confirmed by EDX, which showed a nearly uniform distribution of magnesium on the NC surface (Figure 5c). We also performed XPS measurements to determine whether the magnesium is incorporated in the NC lattice. The binding energies corresponding to Mg 1s core levels indicate the presence of only one chemical environment for Mg^2+^ in the NCs. The shift of the MgCl_2_ treated sample (1303.6 eV) relative to the pure metal halide (1304.6 eV), demonstrates the presence of a new chemical surrounding, suggesting that Mg is also introduced into the crystal lattice (Figure 5d, and see Figure S7 for more detailed information). In addition, XRD data revealed the incorporation of magnesium into the structure by showing a slight shift at higher angles due to the smaller size of Mg compared to Pb (Figure S9a). The effectiveness of the passivation was determined by conducting a study of PLQY over time, achieving a stable luminescence for more than six months (Figure 5e and S9b). Therefore, we can conclude that the origin of these improvements concerning the stability and optical features of the CsPbCl_3_ NCs can be attributed to the dual-surface passivation promoted by both chloride and magnesium ions. Moreover, *in situ* passivation proves to be a more effective, convenient, and cost-efficient (because it reduces the number of steps in the synthesis) approach compared to the post-synthetic method. Nevertheless, the post-synthetic strategy remains promising for repairing NCs damaged due to aging, washing, or dilution. Our optimized synthetic conditions enable the formation of high-quality NCs with long-term stability, paving the way for the development of bright blue emissive halide perovskite NCs for optoelectronic applications.

**Figure 5:**
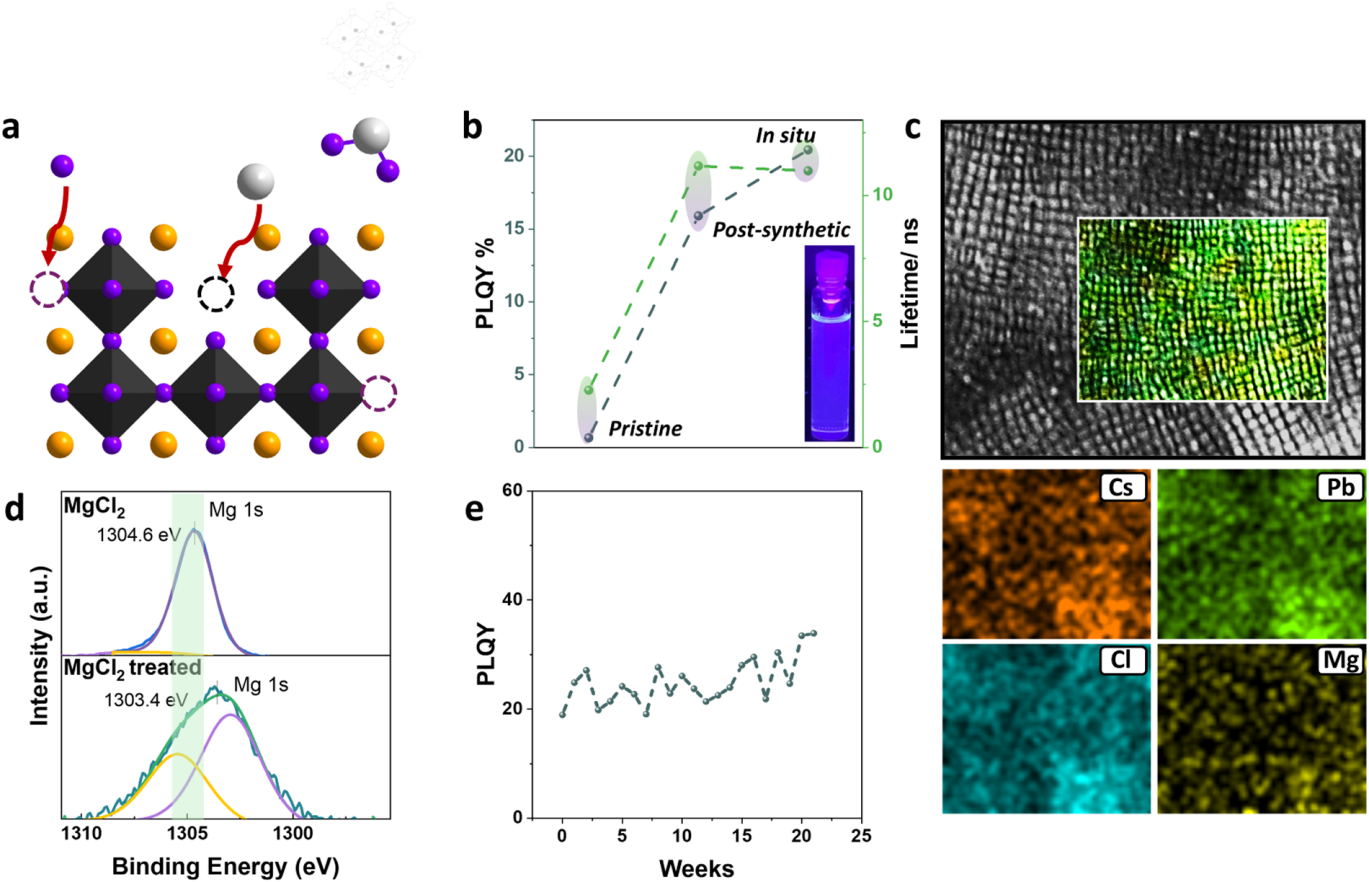
(a) Schematic of the dual surface passivation mechanism proposed for MgCl_2_. (b) PLQY and lifetimes pristine and the best passivation reactant of each kind employed in the postsynthetic surface passivation strategy. (c) STEM image and EDX mappings, where Cs (orange), Pb (green), Cl (blue), and Mg (yellow) are highlighted. (d) XPS measurements of pure MgCl_2_ and CsPbCl_3_–MgCl_2_ treated sample. (e) PLQY over time with CsPbCl_3_–MgCl_2_ treated sample.

To validate the experimental findings of ligand passivation on the CsPbCl_3_ surface,^15,55^ we performed systematic electronic structure calculations corresponding to the adsorption of ligands with three different functional groups (hexylphosphonic acid, hexylsulfonic acid and oleylamine, and tetrabutyl ammonium chloride ligands) on the (001) surface of cubic CsPbCl_3_ perovskites.

The surface structure of CsPbCl_3_ (001) with PbCl_2_ and CsCl terminations are shown in Figure S10­(a,b). The optimized structures of hexylphosphonic acid and hexylsulfonic acid adsorbed on CsPbCl_3_(001) are shown in Figure 6(a, d), while the corresponding projected density of states of the configurations are depicted in Figure 6(c, f). It is worth mentioning here that the phosphonic and sulfonic acid ligands occupy Cl^–^ vacancies while the amine ligands occupy Cs^+^ vacancies on perovskite surfaces.^47^ Hexylphosphonic and hexylsulfonic ligands could form chemical bonds through a single oxygen anchor (single side connection)^20^ or double oxygen anchors (double side connection)^21,56^ on Pb, considering Cl vacancy in the PbCl_2_ terminating surface. On the other hand, oleylamine and tetrabutyl ammonium ligands weakly bind (electrostatic interaction) to the NC surface, occupying the Cs vacancies on perovskite surfaces, as shown in Figure S11­(a,b). Further, the adsorption energies for hexylphosphonic (−1.21 eV) and hexylsulfonic (−0.96 eV) cases are found to be more as compared with the adsorption energies corresponding to oleylamine (−0.65 eV) ligands. Therefore, we can infer that phosphonic and sulfonic group ligands are more favorable as compared to the amine group, as also found in an earlier report.^20^ The electronic structure analysis demonstrates that for the hexylphosphonic acid ligand, the O-*2p* states of the ligands are hybridizing with Pb-*6p*, and therefore will stabilize the halide perovskite surface (Figure 6c). Similar behavior of PDOS has been found for sulfonic ligands, as well (Figure 6f). In the case of ammonium ligand, the corresponding PDOS demonstrates the existence of defect states at the Fermi level, and the presence of midgap states of amine ligands hampers the perovskite surface stability (Figure S11c, d).

**Figure 6:**
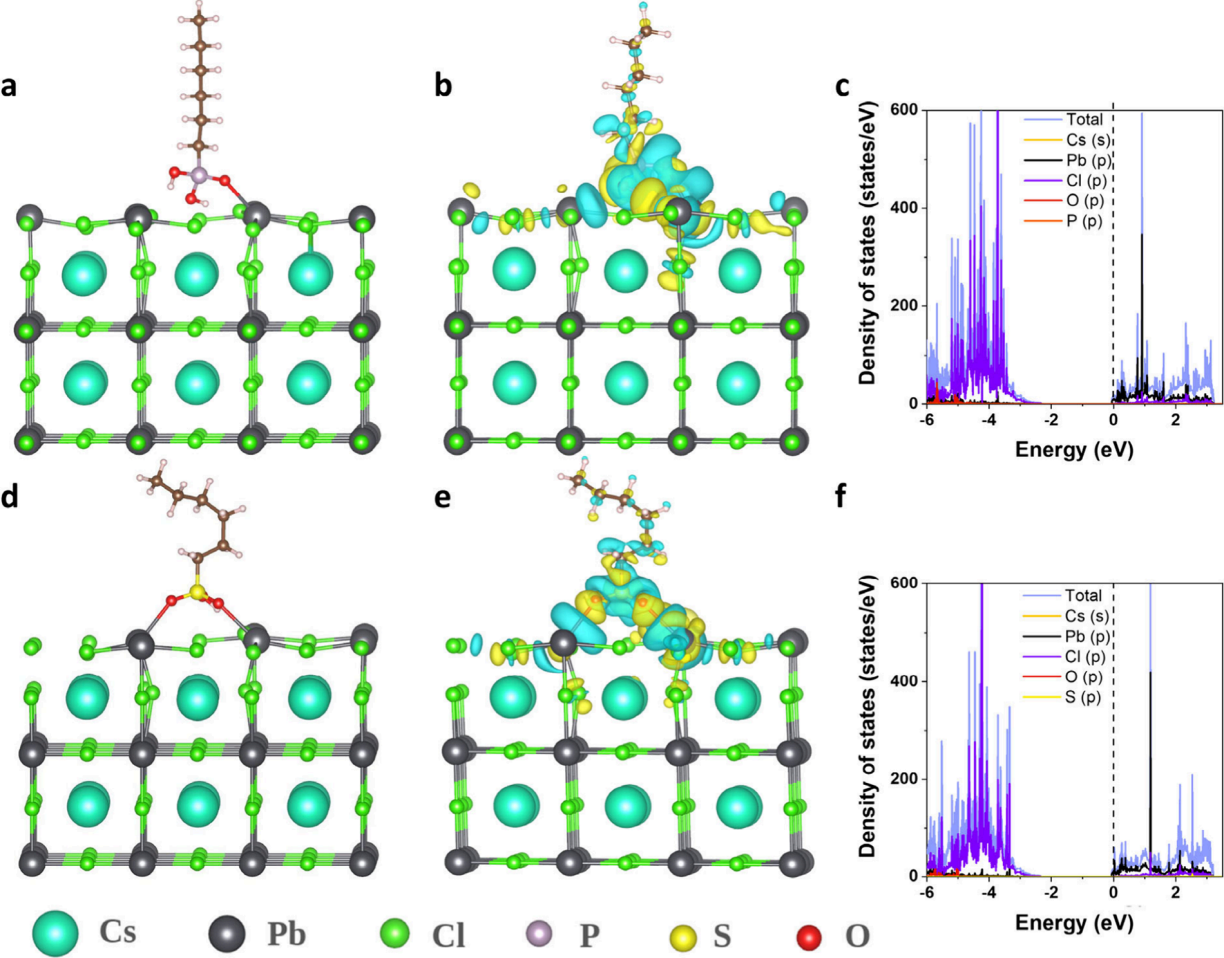
Optimized geometries of (a, d) hexylphosphonic and hexylsulfonic ligands adsorption on PbCl_2_ terminated CsPbCl_3_ (001) surface in the chloride vacancy site, (b, e) charge density difference contour plot for hexylphosphonic and hexulsulfonic, and (c, f) corresponding projected density of states (PDOS) spectra. Fermi energy is shifted at zero.

To have a deeper insight into the ligand bonding with halide perovskite surface, we have determined the charge density distribution and Bader charges to envisage the charge transfer between the perovskite surface and considered ligands for adsorption. As shown in Figure 6(b, e), in the case of hexylphosphonic and hexylsulfonic ligands binding on the halide perovskite surface, the charge transfer is happening from the perovskite surface to oxygen atoms of ligands since the charge is accumulated (yellow contours) around anchoring oxygen and charge is getting depleted (cyan contour) around perovskite surface lead atoms. Whereas for ammonium ligands, there is no charge transfer among surface and ligands atoms (Figure S12) since amine ligands adsorb electrostatically on the perovskite surface. In addition to the charge density distribution, we have reconfirmed our finding through Bader charge analysis regarding the charge transfer from the perovskite surface to ligands through anchoring atoms. In the case of the hexylphosphonic ligand, the Bader charge corresponding to the oxygen atoms of the ligand on the perovskite surface is increased (−1.44e) after adsorption as compared to the pristine case (−1.35e), which confirms the charge transfer from the perovskite surface to the ligands. In the case of sodium hexylsulfonic acid ligand, the Bader charge corresponding to two anchoring oxygen atoms of the ligands on perovskite surface is increased (−1.32e) after adsorption as compared to the pristine case (−1.25e/1.27e), which confirms the charge transfer from perovskite surface to ligands. Additionally, various crystal terminations have been analyzed for the different passivators, as shown in Figures S13–14.

In conclusion, a comprehensive screening analysis of different passivating agents unveiled that phosphonic acid ligands and bivalent metal halides are generally the most promising candidates for effective passivation of deep traps in CsPbCl_3_ NCs and thus yield maximum enhancement in luminescence. Compared to the post-synthetic passivation, incorporating the passivators directly into the precursor solution (in situ passivation) showed improved results, particularly with MgCl_2_, which yielded long-term stable NCs. Furthermore, this study sheds light on the importance of passivating the chloride vacancies, which are highly detrimental to luminescence properties. The electronic structure calculations and the corresponding charge transfer analysis strongly support the experimental finding of the ligand binding on CsPbCl_3_ NCs. These results are encouraging in terms of developing blue emissive NCs with high PLQYs and long-term stability, opening the window to efficient blue LEDs, a goal that has remained challenging. We strongly believe that these findings are not limited to CsPbCl_3_ NCs but are also applicable to other perovskite systems.

## Supplementary Material



## Notes

Detailed overview of additional experimental methods and data including nanocrystal synthesis, STEM characterization, XRD, and computational methodology (PDF)
