# Supplementary material for: Unlocking Brightness in CsPbCl3 Perovskite Nanocrystals: Screening Ligands and Metal Halides for Effective Deep Trap Passivation
Source: ACS Energy Lett. 2025 Mar 12;10(4):1623–32. doi: 10.1021/acsenergylett.5c00185 (PMC12131212; doi:10.1021/acsenergylett.5c00185)
Supplement: Supplementary file 1 [file nz5c00185_si_001.pdf]

Supporting Information for

Unlocking Brightness in $CsPbCl_3$ Perovskite Nanocrystals: Screening Ligands and Metal Halides for Effective Deep Trap Passivation

Nadesh Fiuza-Maneiro,[†] *Junzhi Ye,*[‡] *Shilendra Kumar Sharma,*[#] *Sudip Chakraborty,*[#*] *Sergio Gómez-Graña,*[†*] *Robert L. Z. Hoye,*[‡] *Lakshminarayana Polavarapu*[†*]

[†]CINBIO, Universidade de Vigo, Materials Chemistry and Physics Group, Department of Physical Chemistry, Campus Universitario Lagoas-Marcosende, 36310 Vigo, Spain

[‡] Inorganic Chemistry Laboratory, University of Oxford, South Parks Road, Oxford OX1 3QR, United Kingdom

[#] Materials Theory for Energy Scavenging (MATES) Lab, Department of Physics, Harish-Chandra Research Institute (HRI) Allahabad, A C.I. of Homi Bhabha National Institute (HBNI), Chhatnag Road, Jhunsi, Prayagraj, 211019, India

***Corresponding Author**

Lakshminarayana Polavarapu, Email: lakshmi@uvigo.gal

Sergio Gómez-Graña, Email: segomez@uvigo.gal

Sudip Chakraborty, Email: sudipchakraborty@hri.res.in

Contents:

Supplementary Information 1. Experimental section

Supplementary Information 2. $CsPbCl_3$ characterization

Supplementary Information 3. STEM Characterization

Supplementary Information 4. Absorption spectra after passivation and reproducibility

Supplementary Information 5. PLQY and Lifetime enhancement

Supplementary Information 6. Fluence dependence lifetime

Supplementary Information 7. XPS characterization

Supplementary Information 8. FTIR Characterization

Supplementary Information 9. XRD Characterization and reproducibility PLQY over time measurement of $MgCl_2$ *in situ* passivation sample

Supplementary Information 10. Density of States (DOS) calculations

Supplementary Information 1. Experimental section

Materials: Benzoyl chloride ACS reagent 99%, bis(2,4,4-trimethylpentyl)phosphinic was purchased by Fluorochem 90%, calcium chloride 99.99%, cesium (I) carbonate 99% ReagentPlus® 99%, cesium chloride ReagentPlus® 99% , cyclohexane ACS reagent ≥99% and 9,10-diphenyl anthracene 97% were purchased by Sigma-Aldrich, Didodecyldimethylammonium chloride ≥95% was purchased by Santa Cruz Biotechnology, dimethyldioctadecylammonium chloride 98% was purchased by Fluorochem, 4-dodecylbenzenesulfonic acid 95% was purchased by Sigma-Aldrich, 1-dodecylphosphonic acid 95% from Thermo Scientific, ethyl acetate 99.5 %, hexylphosphonic acid 95%, lithium chloride ReagentPlus® 99%, magnesium chloride anhydrous 98% , 1-octadecene technical grade 90%, , octylphosphonic acid 97% oleic acid technical grade 90%, oleylamine technical grade 70% , potassium chloride ReagentPlus® 99% were purchased by Sigma-Aldrich, sodium 1-hexanesulfonate 99% was purchased by Thermo Scientic, tetrabutylammonium chloride 97%, trioctylphosphine oxide (TOPO) 99%, trioctylphosphine (TOP) 97%, toluene 99.5%, toluene-d8, 99% D and tridodecylmethylammonium chloride 98% were purchased by Sigma-Aldrich.

Preparation of precursors solutions:

Preparation of caesium oleate precursor solution (CsOL). The caesium precursor solution was prepared by dissolving 407.0 mg of caesium(I) carbonate (1.25 mmol) in 20 mL of octadecene with 1.25 mL of oleic acid under strong stirring at 130 °C. The acid-base reaction between the salt and the acid leads to a white precipitate that is only dissolved at temperatures higher than 120 °C, so the precursor must be heated before use.

Nanocrystals synthesis:

$CsPbCl_3$ NCs synthesis

Nanoparticles were synthesized by a hot-injection approach, proposed by *L. Protesescu et al.*[1] First, the lead precursor solution was prepared by dissolving 58 mg of lead (II) chloride (0.20 mmol) in 5 mL of octadecene with the aid of the ligands (0.5 mL of each oleylamine and oleic acid) under strong stirring at 125 °C. Once the solution was dissolved we raised the precursor solution temperature to 180 °C and 0.4 mL of the previously heated cesium oleate precursor prepared as described above was swiftly injected under vigorous stirring. After 3 to 5 seconds, the reaction solution was quenched using a water bath. The as-such synthesized $CsPbCl_3$ NCs were purified in a two-step procedure: first, the solution was centrifuged at 10000 rpm for 10 minutes and redispersed in 3 mL of toluene, and then, the sample was centrifuged at 4000 rpm for 10 minutes to precipitate secondary bulk material formed while supernatant with NCs is conserved.

$CsPbCl_3$ OPA and $MgCl_2$ treated NCs synthesis

For the direct synthesis of the OPA and $MgCl_2$ -treated samples, a similar procedure to that used for the $CsPbCl_3$ NCs was employed, adding 0.14 mmol of each in the synthesis prior to the injection of CsOL.

Preparation of the ligands solutions:

Preparation of the ligands solution. A 20 mM solution was prepared dissolving 0.04 mmol of the ligand in 2 mL of toluene. Then, a 2 mM solution was prepared by dilution of the previous one.

Optical Characterization

UV-Vis extinction spectra were obtained using a Cary 8454 spectrophotometer (Agilent). Photoluminescence spectra were obtained with a Cary Eclipse Fluorescence Spectrophotometer (Agilent).

PLQY measurements were performed through a standard relative method employing a procedure previously reported[2-4] using 9,10-Diphenylanthracene dissolved in cyclohexane as a reference dye acquired using an excitation wavelength λex = 350 nm for all of the measurements. All solutions were diluted to an optical density of 0.1 or lower at the excitation wavelength to minimize reabsorption processes. Quartz cuvettes with an optical path length of 1 cm were used for all optical measurements.

Determination of the Fluorescence Quantum Yield (Φf)

The fluorescence quantum yield (Φf) of a fluorophore can be determined using a relative optical method, which involves the following sequential steps:

1. **Spectral Measurements**: The absorption and emission spectra of the sample are recorded.

2. **Selection of a Fluorescence Quantum Yield Standard**: A suitable reference fluorophore with a well-characterized fluorescence quantum yield is selected. The standard should exhibit absorption and emission properties within a similar wavelength range as the sample (**Figure S1**). Furthermore, the quantum yield of the reference dye should be well established under the same experimental conditions, including solvent or matrix composition, excitation wavelength, temperature, and chromophore concentration. In our case, we have selected 9,10-Diphenylanthracene dissolved in cyclohexane whose broadband luminescence centred in the deep-UV region correlates with the emission of $CsPbCl_3$ NCs.

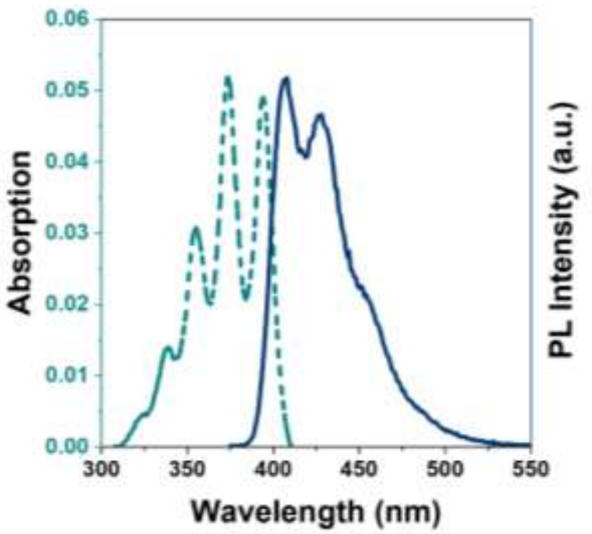

Figure S1. PL (solid line) and absorption (dotted line) spectra of the standard reference dye 9,10-diphenylantracene in cyclohexane.

3. **Experimental Setup and Measurement Conditions**: Measurement parameters such as the excitation wavelength (λ_{ex}) and absorbance at λ_{ex} are carefully chosen. The same instrument settings must be applied for both the sample and the standard to ensure comparability.

4. **Acquisition and Background Correction**: The absorption and emission spectra of both the sample and the standard are recorded. Additionally, the emission spectra of the corresponding solvents are measured and subtracted from the sample and standard spectra to eliminate background signals arising from solvent fluorescence, light scattering, and dark counts at the detector. In our case, background correction is not necessary as toluene and cyclohexane emission properties are shifted and their intensity is so low that it is not appreciable under our measurement conditions.

5. **Calculation of the Relative PLQY**

The relative PLQY (Φf) has been determined through the use of Equation 1 after the data processing. In this equation, Φf and Φf_{Dye} represents the photoluminescence quantum yield of the sample and the reference fluorophore respectively. The parameter "m" represents the slope of the linear regression obtained from the plot of absorbance versus integrated area of fluorescence intensity. In addition, the equation includes a refractive index correction term "n", where n_{Sample} is the refractive index of the solvent ($n_{toluene}$ =1496) and n_{Dye} is the refractive index of the reference or dye ($n_{cyclohexane}$= 1427). Furthermore, on the other hand, the Φf_{Dye} was referenced in the equation taking into account the value previously proposed in literature for this solvent at the corresponding excitation wavelength (Φf_{Dye} =1).[2]

$$\Phi f_{Sample} = \frac{m_{Sample}}{m_{Dye}} \cdot \Phi f_{Dye} \cdot \left(\frac{n_{Sample}}{n_{Dye}}\right)^2 \qquad \text{Eq. 1}$$

This method ensures accurate determination of fluorescence quantum yield by minimizing instrumental and environmental influences while maintaining reliable comparability between the sample and reference standard.

Scanning Transmission Electron Microscopy (TEM) Characterization

Scanning transmission electron microscopy (TEM) images were obtained with a JEOL JEM 2010F field-emission gun TEM (JEOL Corporation, Akishima, Tokio, Japan) operating at 200 kV. High Resolution TEM Images (HRTEM) were collected with a JEOL JEM F200CF-HR microscope (200 keV) with a cold field emission gun (COLD FEG) and an HR objective lens. Images were acquired at 120 kV.

Nuclear Magnetic Resonance (NMR) Characterization

^{31}P-NMR spectra were recorded in toluene-d8 at 298 K with a Bruker AMX-400 spectrometer operating at 162 MHz. ^{31} P NMR spectra in toluene-d8 were recorded with 10104 scans and a relaxation delay of 4 s over a spectral width of 280.5833 ppm.

Sample Preparation

For the NMR characterization, the samples were dried using a N_2 flow and redispersed in deuterated toluene (300 μL).

XPS Characterization

The samples were prepared by drop-casting 300 μL NC solutions (similar concentration) onto spectroscopic glass substrates. Once dried, the films were sent for XPS measurement.–XPS data was acquired using a Thermo Fisher Scientific K-Alpha with a spherical sector and a multichannel resistive plate detector with 128 elements. The excitation source was an Al Ka monochromatic (1486.6 eV) with a constant pass energy through a multichannel direct signal mode. High-resolution spectra were obtained using a pass energy of 20 eV, step size of 0.1 eV. Survey spectra were obtained using a pass energy of 200 eV, with an energy step size of 1 eV.

FTIR Characterization

The FTIR data were collected employing a Nicolet 6700 spectrometer (Thermo Fisher). The samples were deposited on previously synthesized KBr pellets and measured once dried.

.X-Ray Diffraction

XRD analysis was performed using a PANalytical -XPERT-PRO X-Ray diffractometer. XRD patterns were obtained equipped with a 1.54 kW Cu Kα, operating at 40 kV and

30 mA. The samples for the XRD measurements were prepared by drop-casting a concentrated NC solution into a glass cover slide.

Time-resolved photoluminescence lifetime

The laser employed is a Coherent Mira 900 Ti:Sapphire laser. An Inrad 5-050 ultrafast harmonic generation system is used to generate the 350 nm wavelength laser. The second harmonic is used as the excitation source for PL. The repetition rate is 7.6 MHz, and the beam used had a 1 μm radius. All samples were prepared by drop-casting the solutions.

The tri-exponential decay fit is selected to phenomenologically model the PL decay curve so that we can quantitatively compare NCs treated with different passivators. We believe that the tri-exponential model itself does not directly represent any physical process of the charge-carriers besides providing a numerical description. Fitting a model with physical significance requires careful collection of much more information going beyond simply measuring the TRPL decay curve. The reason we choose the simplest numerical fitting model over other models is because of the relatively complicated radiative species in $CsPbCl_3$ NCs. Since the size of the NCs we prepared is around 6 nm, which is similar to the exciton Bohr radius of the material (~ 5 nm),[5] the radiative emission may come from a mixture of free carriers and excitons, which makes the fitting using the other models inaccurate. Since, we are interested in only comparing the average lifetime for NCs with different ligands, a simple tri-exponential would be sufficient. Also, we found that fitting with numerical method and physical model (account bulk and surface recombination) method do not vary the fitted average lifetime too much.[6]

Supplementary Information 2. $CsPbCl_3$ characterization

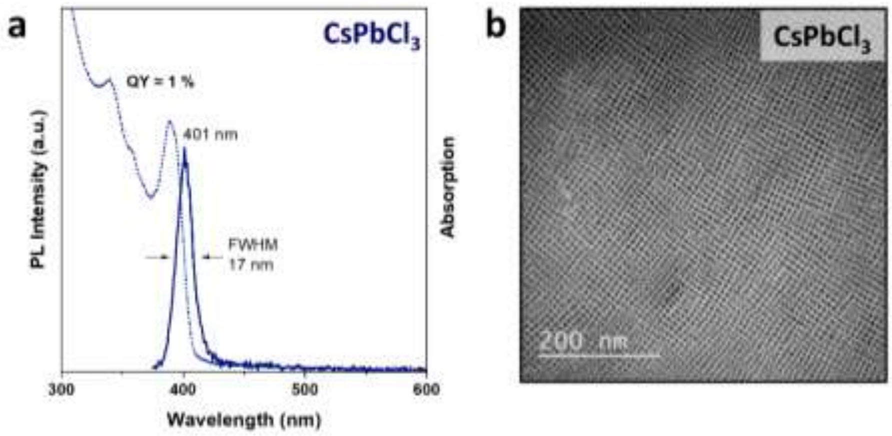

Figure S2. a) PL (solid line) and absorption (dotted line) spectra of $CsPbCl_3$ NCs. b) HRTEM image for $CsPbCl_3$ NCs.

Supplementary Information 3. STEM Characterization

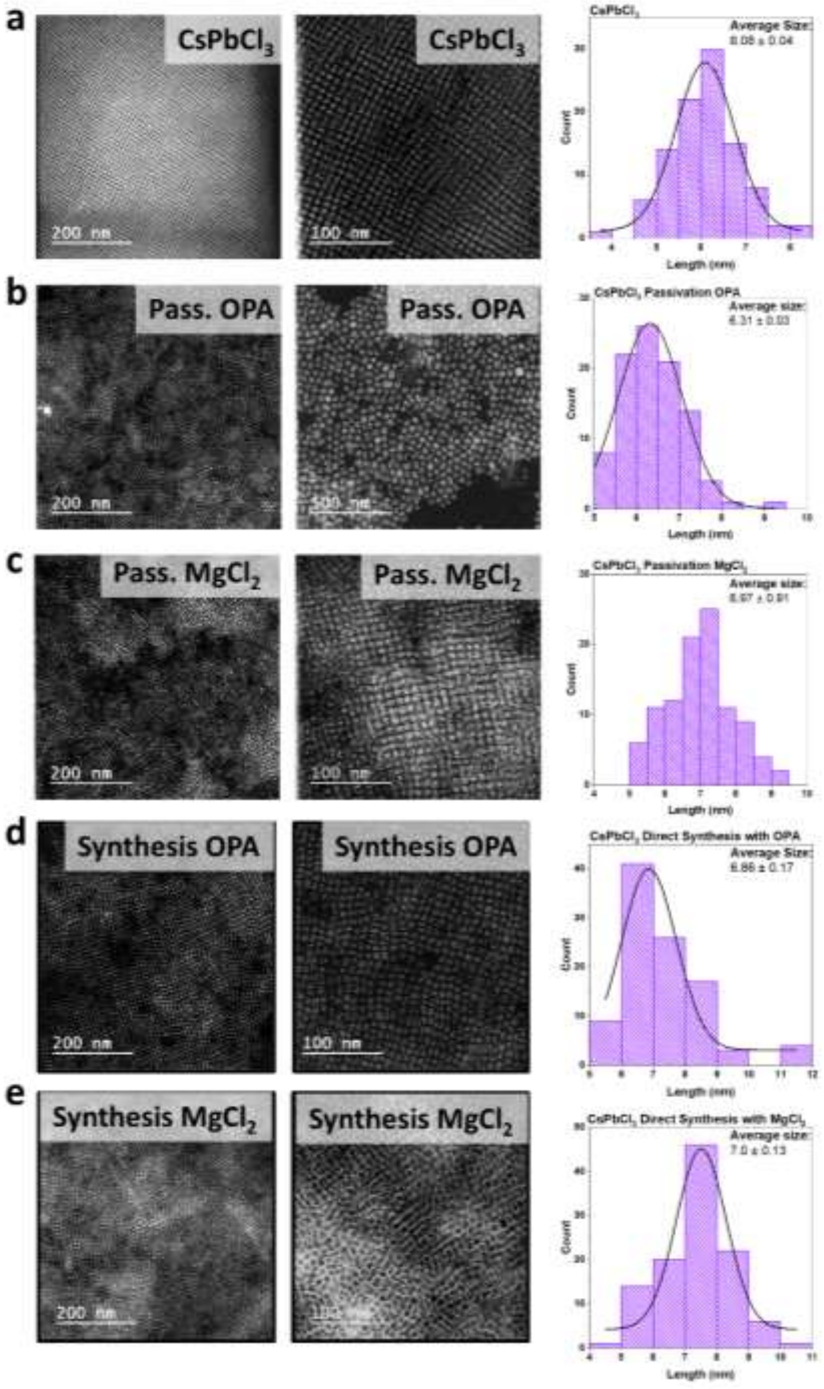

Figure S3. STEM and Histogram of a) $CsPbCl_3$ NCs and $CsPbCl_3$ passivated NCs with b) OPA and c) $MgCl_2$, and d) Direct synthesis with OPA and e) $MgCl_2$ NCs.

Supplementary Information 4. Absorption spectra after passivation and reproducibility

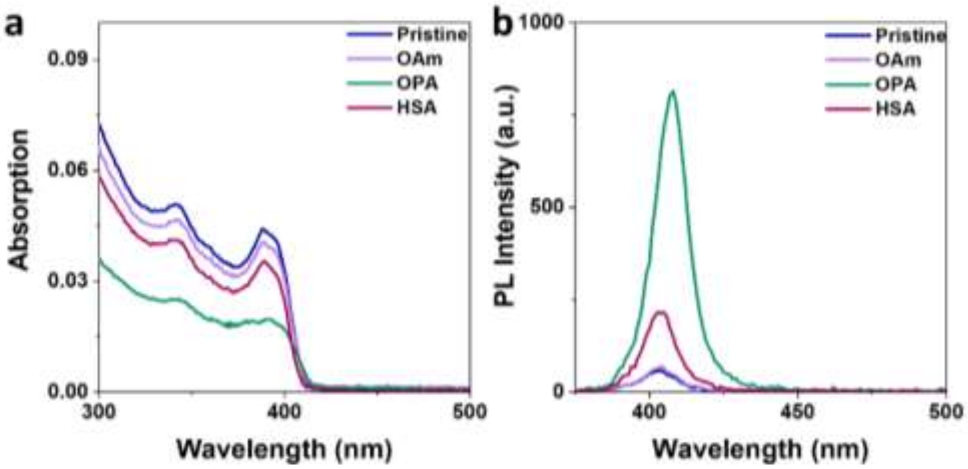

Figure S4. a) $CsPbCl_3$ NCs and $CsPbCl_3$ passivated NCs absorption spectra. b) PL Intensity spectra of $CsPbCl_3$ NCs and $CsPbCl_3$ passivated NCs spectra.

Supplementary Information 5. PLQY and Lifetime enhancement

In the following table, some of the reactants used in the passivation are shown together with the improvement factor and lifetimes obtained.

Table S1. PLQY enhancement factor and time-resolved PL decays (exponential fitted) (power 10µW) of $CsPbCl_3$, NCs after treatment with passivating reactants.

10µW	A_1	τ_1	A_2	τ_2	A_3	τ_3	τ_{ave}	PLQY factor
**Pristine**	665.6	0.1	35.2	1.0	18.9	5.0	2.2	1
**OPA** pass	483.6	0.2	84.7	1.3	35.3	9.0	5.9	9.6 ± 3.0

**OPA Direct**	466.3	0.2	139.8	7.3	2.3	26.0	7.7	14.8 ± 1.5
**$MgCl_2$ pass**	479.7	0.3	61.9	4.2	66.9	14.9	11.1	20.5 ± 2.6
**$MgCl_2$ Direct**	502.8	0.2	111.5	8.3	15.0	22.0	10.9	22.8 ± 4.5
**HSA_{pass}**	502.8	0.2	122.3	1.0	27.2	7.4	3.9	3.8 ± 0.6
**OAm_{pass}**	599.5	0.1	51.7	1.2	11.3	8.0	3.4	1.3 ± 0.1
**HPA**	32354.2	0.1	8575.0	1.1	1026.1	10.0	4.4	9.1 ± 1.1
**DPA**	51.0	0.1	5.9	2.0	1.0	10.0	4.2	8.1 ± 1.9
**Bis**	101.1	0.1	11.6	1.0	854.0	13.0	4.2	3.9 ± 0.3
**DBSA**	2384.0	0.2	664.1	1.2	86.1	9.0	3.9	2.0 ± 0.2
**DSA**	3322.6	0.1	510.8	2.0	105.0	7.0	3.2	1.5 ± 0.1
**$CaCl_2$**	37483.2	0.4	9594.0	2.2	1604.4	12.0	5.2	11.6 ± 1.5
**Benzoyl chloride**	3.3	0.2	488.0	1.0	35.0	15.0	4.7	6.9 ± 0.1

In addition to the passivating agents listed in the table, the post-synthetic procedure has also been applied to a large number of other reagents which, due to their low or null effectiveness, have not been characterized by PLQY or lifetime, such as: 3-(decyldimethylammonio)-propane-sulfonate inner salt, didodecyldimethyl ammonium chloride, dimethyldioctadecyl ammonium chloride, dodecyltrimethyl ammonium chloride, tetrabutyl ammonium chloride, tetraoctyl ammonium chloride, dimethyl methyl phosphonate, trioctylphosphine, trioctylphosphine oxide, octanoic acid, lithium chloride, potassium chloride, cesium chloride, sodium chloride and miltefosine.

Supplementary Information 6. Fluence dependence lifetime

To make our TRPL data more clear to the readers, we have added the fluence-dependent data with the instrument response function (IRF). In our fitting parameters, the reason for the initial fast decay is due to the overlap with the IRF, and this initial decay is also nearly fluence independent. However, we believe the short decay component is not due to trap-related process in our case. **Figure S5f** overlays the PL decay with the decay obtained from the transient absorption spectroscopy measurements. We can see there is a fluence independent fast drop in the carrier density within the first 10 ps after photoexcitation, which is much faster than the carrier trapping process for perovskite (trapping usually takes place on the ~100 ps timescale). We also noticed that this fast decay in TA is shorter than the time resolution of TRPL measurement, which explains why we would observe the IRF to be visible. At the 10 ps timescale, considering the high fluence used (larger than 40 μJ cm^2), the recombination process is likely dominated by Auger or exciton-exciton annihilation. Thus, the longer time constants fit

to the PL decay over the ~100 ps timescale are likely more related to radiative and trap-mediated recombination processes, which both exhibit strong fluence dependence in both the TA and TRPL measurements. The data shows that there is more clear change in TRPL decay for the pristine NCs than the OPA and $MgCl_2$ treated samples, indicating that the passivation works better when using these two types of passivators. This is aligned with our observations in PLQYs.

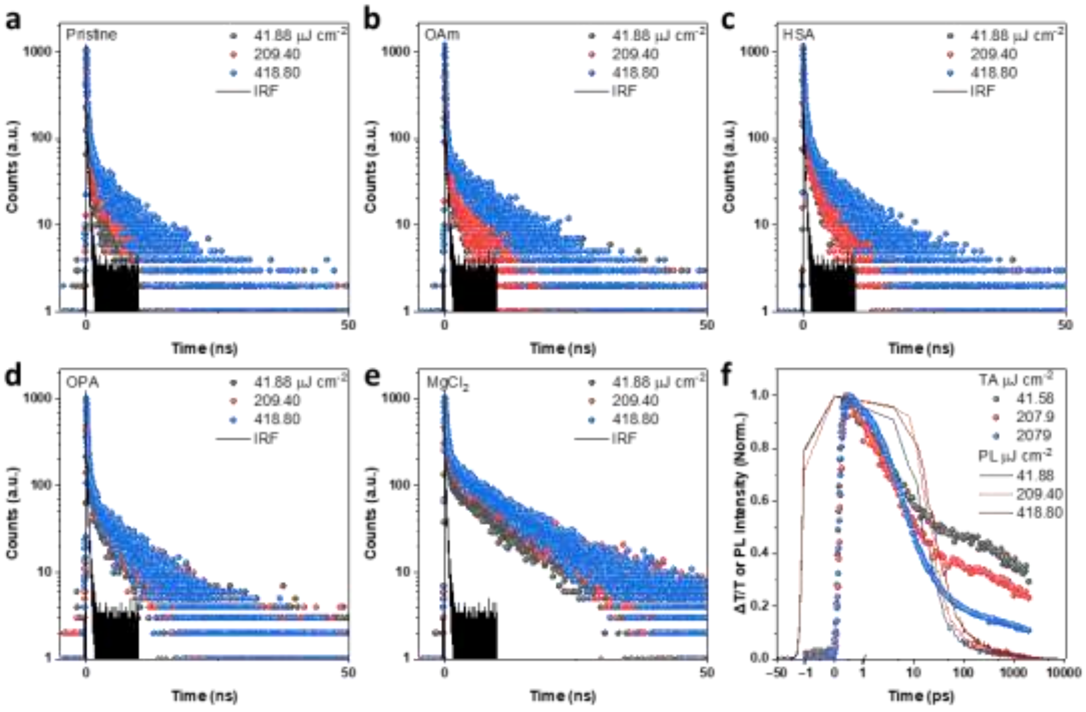

Figure S5. Fluence dependance data with the internal response function (IRF) for a) Pristine, b) Oleylamine, c) HSA, d) OPA and e) $MgCl_2$ passivated samples and f) PL decay with the decay obtained from the femtosecond transient absorption spectroscopy measurements.

Supplementary Information 7. XPS characterization

To determine whether the sodium from the sulfonate is passivating the surface, we analyzed the XPS spectra of each sample to approximate the Na1s peak position, referencing the adventitious carbon at 285 eV. In the HSA treated sample, we detected a significantly lower sodium concentration compared to Sodium Hexanesulphonate, as evident in the comparative spectra. The Na1s peaks of both samples are nearly overlapping, with binding energies of 1071.1 eV (HSA) and 1071.3 eV (HSA treated), a minimal difference that should not be attributed to sodium incorporation into the perovskite lattice. Instead, this slight 0.2 eV shift likely results from the low sodium concentration in the HSA treated sample, leading to minor binding energy indeterminacy. We attribute the minimal sodium detected in HSA treated sample to residual traces not completely removed during purification.

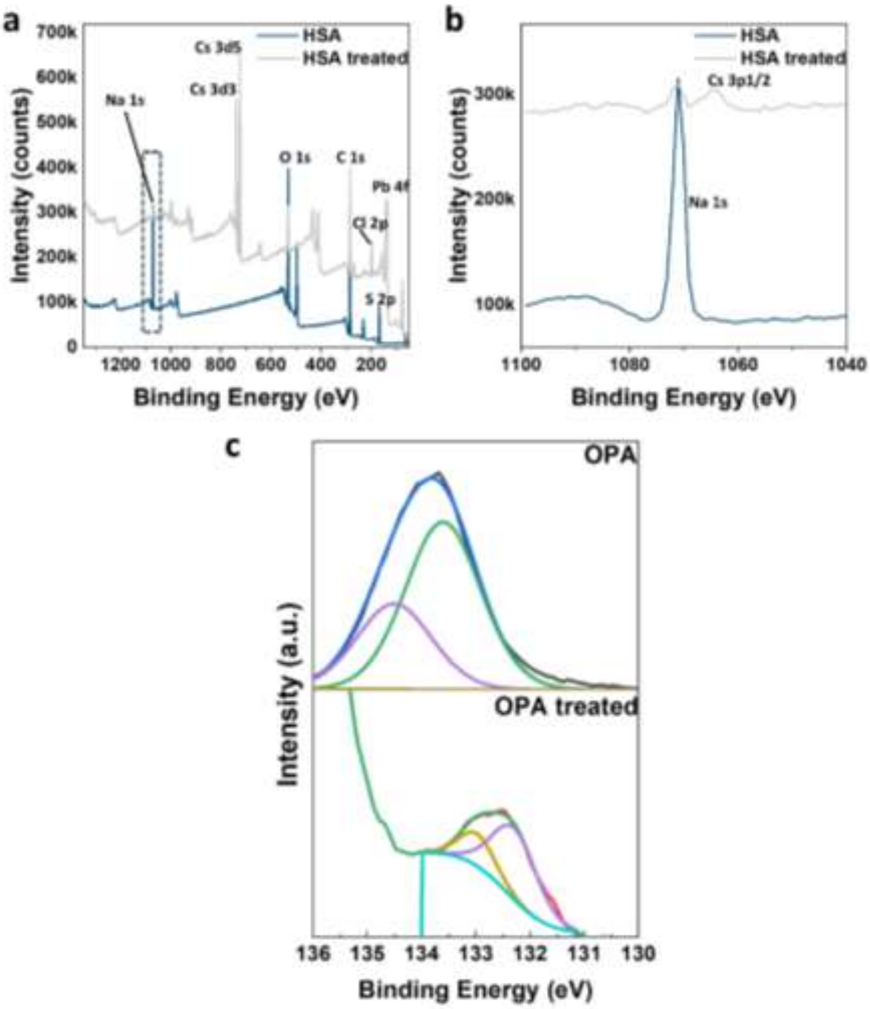

Figure S6. a, b) XPS survey spectra for HSA treated sample. c) XPS results for free OPA ligand and OPA *in situ* passivation treated sample.

In the case of the XPS measurements of $MgCl_2$, we were especially meticulous in order to avoid confusion with any other magnesium species. By measuring the metal halide ($MgCl_2$), we accurately determined the binding energies of Mg and Cl in this compound, reaching a stoichiometric ratio of 0.53 Mg:Cl, practically identical to the theoretical value. The same accuracy was observed in pristine perovskite, where we found an almost perfect ratio of 1:1.01:3.08 (Pb:Cs:Cl). In addition, we successfully extracted the binding energies of all key elements, including oxygen.

In order to rule out the presence of magnesium oxide, we carried out successive analyses on the basis of different elements as described below:

C1s Functional Groups: The analysis of the C1s peak revealed the presence of functional groups in this element, including both single (C–O) and double (C=O) bonds. These are likely originated from oleic acid and other organic contributions, which justify and necessitate the detection of oxygen.

Binding energy of O1s: The O1s binding energy in both pristine perovskite and $MgCl_2$-treated perovskite is nearly identical. In pristine perovskite, a single symmetric O1s peak appears at 531.87 eV, which we attribute to the oxygen from the oleic acid. In the case of the modified perovskite with $MgCl_2$, the O1s peak shifts only slightly to 531.7 eV, confirming that the oxygen originates from oleic acid functional groups and not from the formation of oxides or hydroxides.

To confirm this, we also searched the literature for O1s binding energy values when oleic acid is used as a surfactant and synthesis agent in various materials and applications to compare with our own data.

The binding energies referenced range from: 531.2-531.6 eV

1) 531.2 eV (Single peak)[7]

2) 531.3 eV f(Coexisting with peaks at lower energies of metal oxides)[8]

3) 531.6 eV carboxylic bonds from oleic acid coexisting with peaks at lower energies of metal oxides.[9]

4) 531.6 eV from C-O and C=O in oleic acid.[10]

These reported BE for O1s fall in the range of our values, which makes us more confident in the assignment of that energy.

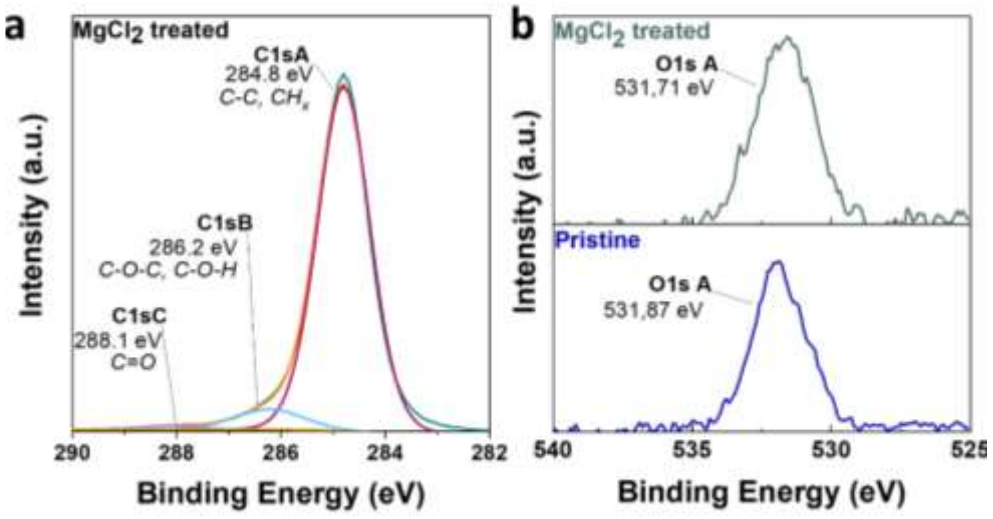

Figure S7. a) C1s XPS spectra of $MgCl_2$ treated sample. b) O1s XPS spectra comparison of both pristine and $MgCl_2$ treated sample.

Magnesium peak Mg1s: A direct comparison shows that the binding energy (BE) of the Mg1s peak in $MgCl_2$-treated perovskite does not match that of reference $MgCl_2$ (See **Figure 5d**). Instead, the Mg1s peak envelope shifts to lower energies. Specifically, the Mg1s peak maximum in $MgCl_2$ appears at 1304.6 eV, while in the modified perovskite, it shifts to 1303.6 eV indicating a 1 eV shift toward lower energies.

For the case of Mg1s, a direct comparison between the BE of the $MgCl_2$ (1304.6 eV) and the treated perovskite (1303.6 eV) shows a clear shift. By examining the expected binding energies for the corresponding oxides, MgO (1303.9 eV) and $Mg(OH)_2$ (1302.7 eV), we can rule out the hydroxide since it is too displaced. However, MgO is close in binding energy to the value experimentally reported for the sample. Nevertheless, the substitution of Cs^+ or Pb^{2+} cations by Mg^{2+} would result in a shift to lower BE, as observed (1303.6 eV), due to the decrease in atomic number, reducing the nuclear charge and consequently, the binding energy of the electrons, which experience a weaker attractive force.

Supplementary Information 8. FTIR Characterization

FTIR measurements revealed a peak at approximately 1000 cm^{-1}, which can be attributed to P–O stretching vibrations. This peak is associated with P–O stretching from phosphonate or phosphonic groups, as well as potential P–OH bending modes. It is important to note that slight shifts in this peak may occur due to variations in the chemical environment, hydrogen bonding, and the degree of protonation.

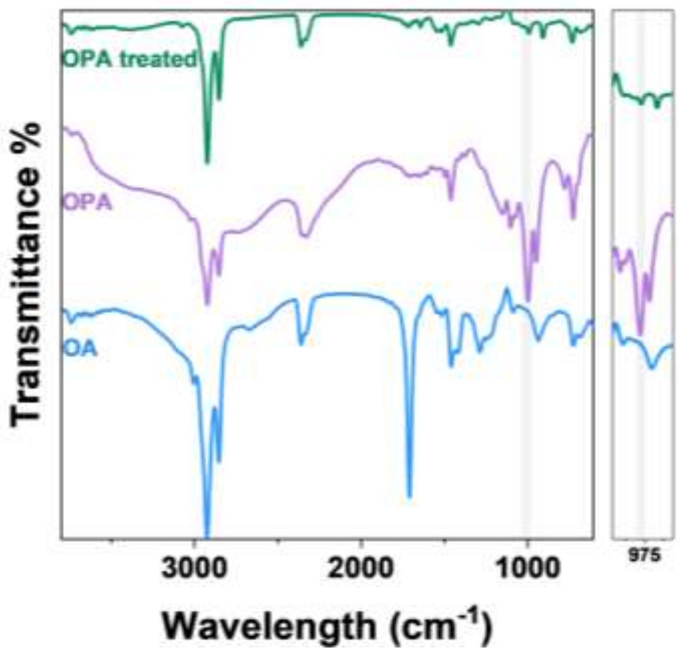

Figure S8. FTIR spectra of OPA treated sample and OPA and OA ligands.

Supplementary Information 9. XRD Characterization and reproducibility PLQY over time measurement of $MgCl_2$ *in situ* passivation sample

The graph below shows how the angle in the XRD spectrum of the sample containing Mg^{2+} atoms in the crystal structure is slightly modified. As indicated in the literature, if the XRD peak shifts towards higher angles, it means that the lattice parameter of the crystal has decreased or the crystal structure has contracted, which corresponds to our results where the peak of the $MgCl_2$ sample shifts towards higher angles due to the smaller size of the magnesium ionic radio (0.065 nm) concerning the lead (0.119 nm) or the cesium (0.169 nm).

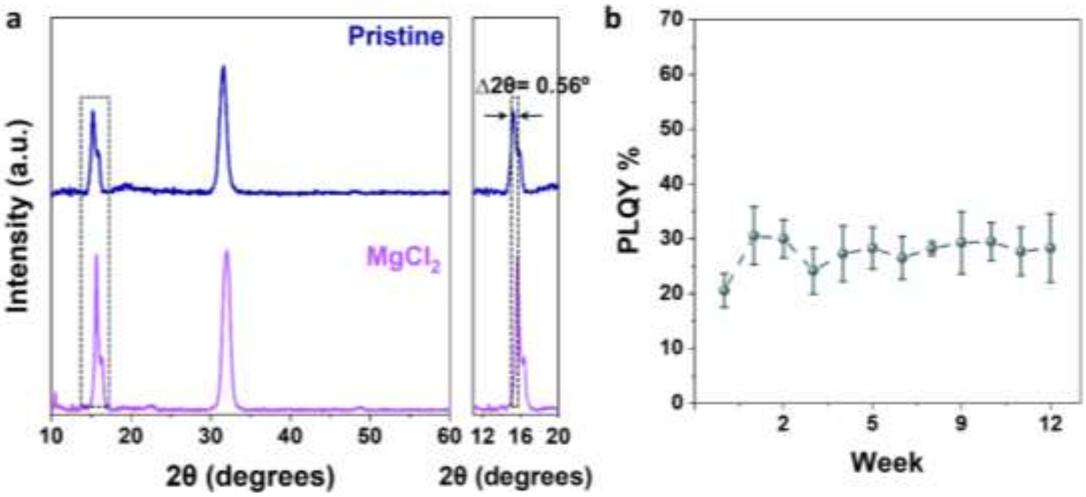

Figure S9. a) XRD spectra of Pristine and $MgCl_2$ *in situ* passivation sample. b) PLQY reproducibility over time of *in situ* passivation of $MgCl_2$ sample.

Supplementary Information 10. Density of States (DOS) calculations

Quantum based density functional theory (DFT) electronic structure calculations were performed to study the effect of ligands on $CsPbCl_3$ perovskite surface [11, 12]. We considered 001 the most stable surface of $CsPbCl_3$ cubic perovskite for ligand absorption. To properly accommodate ligands on the $CsPbCl_3$ surface, we considered a 3x2x1 supercell of 001 cleaved surface with 18 Angs vacuum in z-direction. Perdew Burke Erzernhof (PBE) exchange-correlation functional within the framework of projected augmented wave (PAW) formalism was utilized in all calculations [13, 14]. Vienna *ab-initio* simulation package (VASP)[15, 16] was exploited in all calculations. Plane wave energy cut off 450 eV and a Monkhorst pack scheme 3x4x1 kpoints were utilized for Brillouin zone sampling for geometry optimizaton[17]. Energy convergence and force convergence criteria were kept 10^{-5} eV and -0.01 eV/Å. Structures visualizations and image generation were performed by utilizing VESTA software[18]. All calculations were performed with Grimme dispersion corrected DFT D3 approach to account for van der Waals interaction between 2D layers[19].

The adsorption energy of ligands' adsorption was calculated by utilizing the given formula:

$E_{ads} = E_{total} - E_{surface} - E_{adsorbate}$

E_{ads}= Adsorption Energy of the adsorbate ligand (molecule)

E_{total}= Total energy of the ligand (molecule) adsorption on the considered surface

$E_{surface}$= Energy of the pure surface slab

$E_{adsorbate}$ = adsorbate ligand (molecule) energy

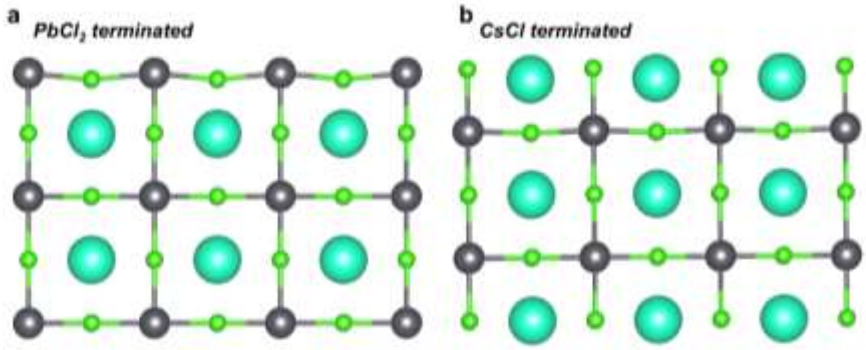

Figure S10. Scheme of $CsPbCl_3$ NCs with (a) $PbCl_2$ and (b) CsCl surface termination where grey atoms correspond to lead, cyan to cesium and green to chloride.

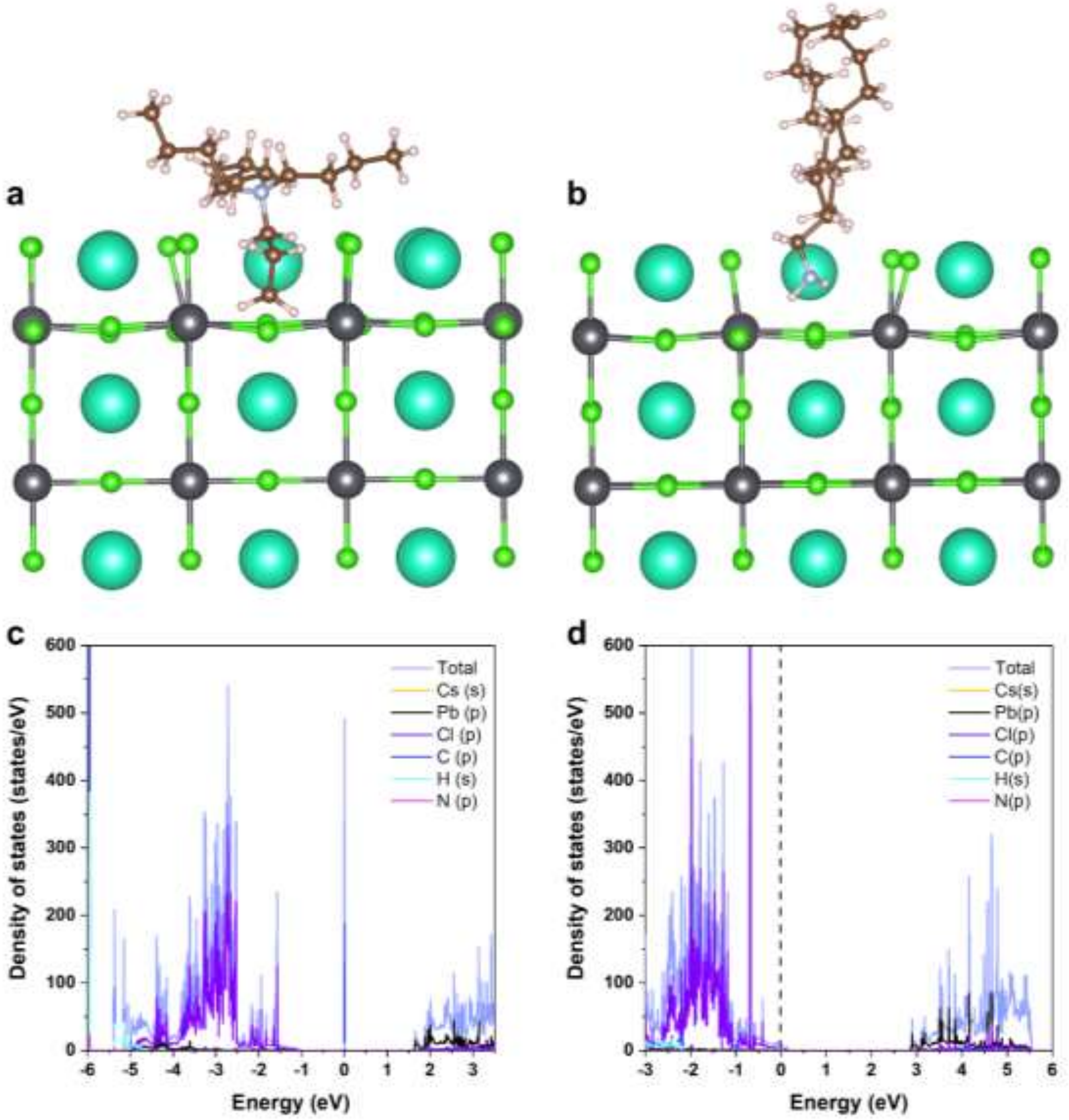

Figure S11. Optimized geometries of (a) tetrabutylammonium and (b) oleylamine ligands adsorbed on perovskite surface in the Cs cation vacancy site, (c) and (d) are corresponding density of states (DOS) spectra.

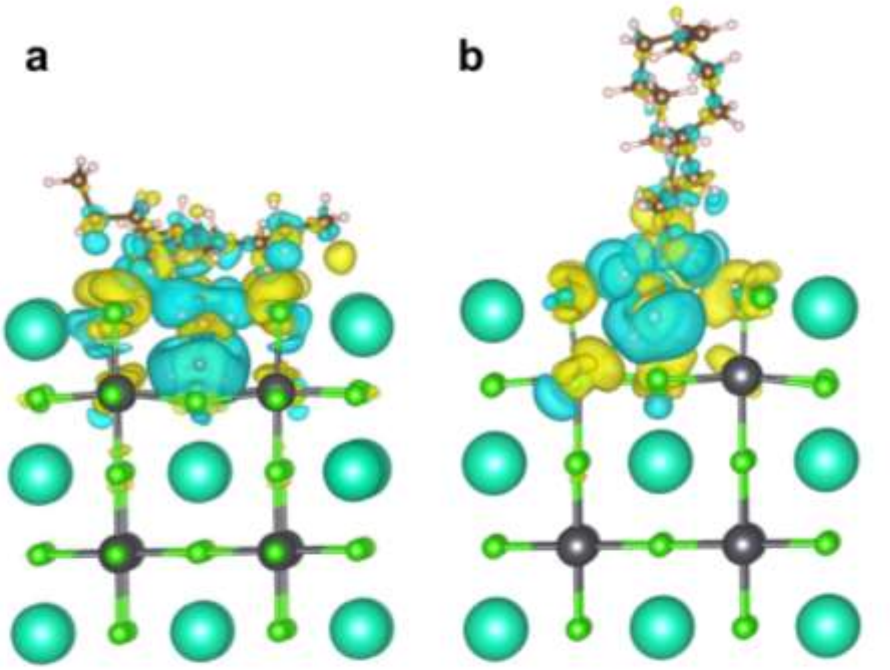

Figure S12. Charge density difference plot of a) tetrabutyl ammonium chloride and b) oleylamine ligands adsorption on perovskite surface.

In addition, we have performed ligand adsorption DFT calculations on (110) surface[20] of $CsPbCl_3$ perovskite (which includes both Pb and Cs terminations simultaneously). The calculations are performed without any surface Cl anion vacancy (unlike previous calculations on 001 surface). Further, we have calculated adsorption energies of all three types of considered ligands (hexylphosphonic acid, sodium hexylsulfonic acid, and tetrabutylammonium chloride) adsorption on (110) surface. **Figure 13a** and **b** represent optimized geometries of (110) surface of $CsPbCl_3$ in two different perspectives, while **Figure 13c, d,** and **e** represent optimized geometries of ligands (hexylphosphonic, hexylsulfonic, and tetrabytylammonium ligands) adsorption on (110) surface of $CsPbCl_3$ perovskite respectively.

Table S2. Adsorption energies for ligands adsorption on $CsPbCl_3$(110) surface

Ligands	Adsorption Energy at 110 surface (eV) Without Cl vacancy	Adsorption Energy at 001 surface (eV) with Cl vacancy (from previous calculations)
**Hexylphosphonic**	-1.09	-1.21
**Hexylsulfonic**	-0.74	-0.96
**Tetrabutylammonium**	-0.68	-0.65

Further, we have found adsorption energies of hexylphosphonic acid (-1.09 eV) is greater than hexylsufonic acid (-0.74 eV) (**Table S2**) anchoring via Pb-O bond for hexylphosphonic while Sulfonic ligand anchor via Pb-O and Cs-O bonds as shown in optimized geometries **Figure 13c** and **d**. On the other hand, tetrabutylammonium ligand adsorption energy was found least (-0.65 eV) which makes physisorption on $CsPbCl_3$(110) surface. Thus, all the ligands adsorption energies trend on (110) surface (without Cl vacancy) is also similar as on (001) surface with Cl surface anion vacancy (see main manuscript and **Table S2**).

It has been found phosphonic and sulfonic ligands adsorption energies are more prominent in case of (001) surface (Pb termination) with Cl anion surface vacancies in comparison to (110) surface (Pb and Cs terminations) without Cl vacancies (**Table S2**). Thus, it can be inferred that phosphonic and sulfonic ligands would like to bind in the vicinity of surface Cl anion vacancies. In the case of tetrabutylammonium ligand adsorption energies for (110) and (001) surface are similar, while tetrabutylammonium

ligand makes physisorption on perovskite surfaces as compared to Phosphonic and Sulfonic ligands which anchor through chemisorption on perovskite surfaces.

We also have calculated projected density of states (PDOS) of (110) surface after ligand adsorptions. **Figure 14a** shows PDOS of phosphonic ligand adsorbed on (110) surface. Pb-6p surface defect states are available in the band gap region below the conduction band minima edge which is good agreement with previous study of YCl_3 dual passivation of $CsPbCl_3$ perovskite.[21] Additionally, the O-2p ligand states are present in the deep valence band region. The surface defects are more prominent at the (110) surface in comparison to the $PbCl_2$ and CsCl terminated (001) surfaces (explained in the main manuscript) with Cl and Cs vacancies respectively. The presence of defect states in the band gap region would decrease the PLQY of the nanocrystal and ligand modification will try to passivate these defects. In conclusion, the (110) surface will show less PLQY due to the presence of more surface defects in comparison to the (001) surface with different cation terminations. Similar behaviour is also observed for Hexylsulfonic ligand PDOS as shown in **Figure 14b.**

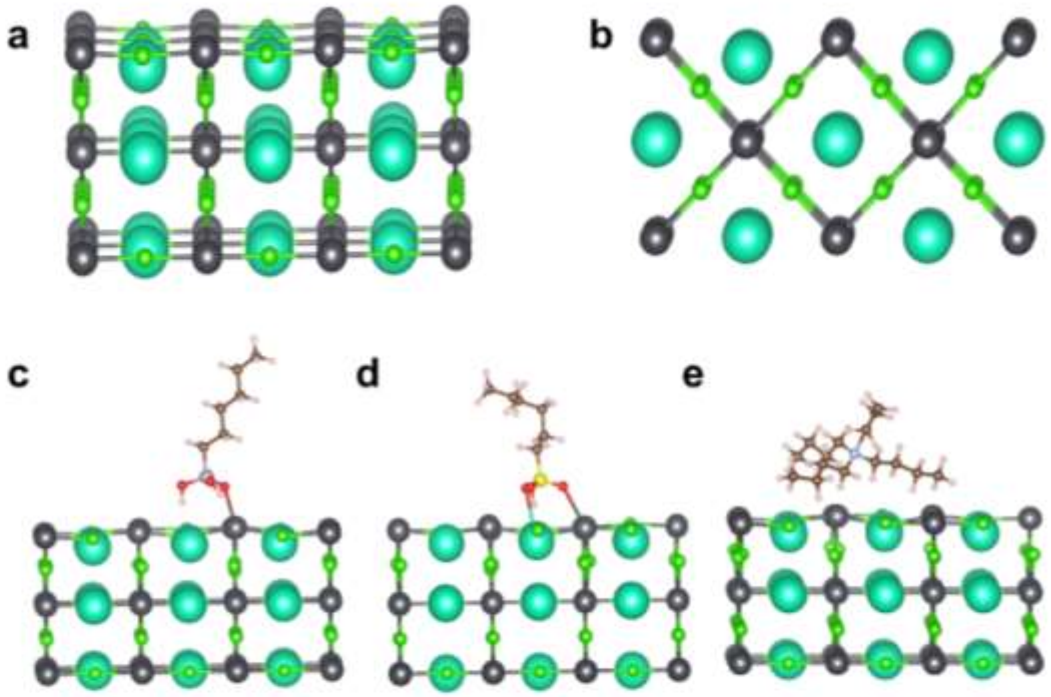

Figure S13. a) and b) are two different perspectives of $CsPbCl_3$ (110) surface with both cation (Pb and Cs) terminations on surface without surface Cl anion vacancies, c) adsorbed hexylphosphonic, d) adsorbed hexylsulfonic, and e) adsorbed tetrabutylammonium ligands on $CsPbCl_3$ (110) surface.

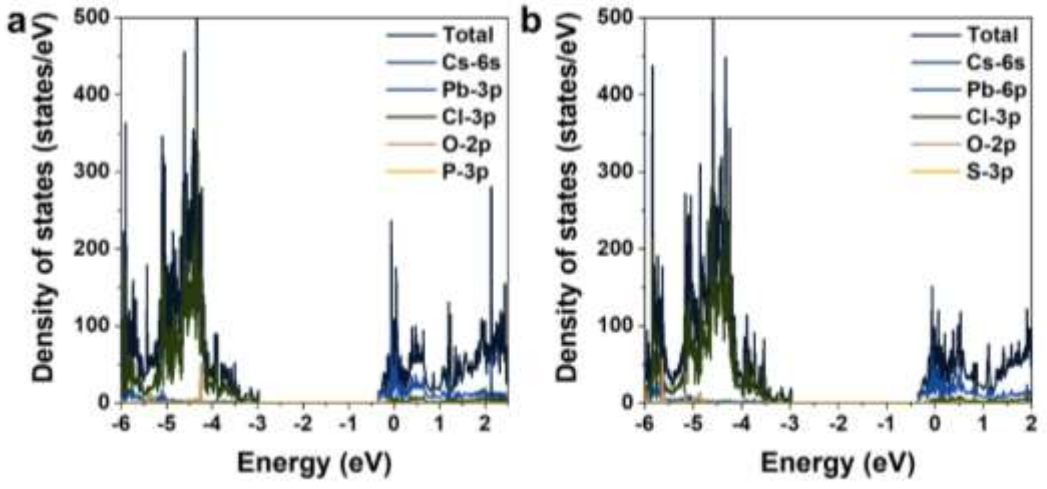

Figure S14. Projected density of states (PDOS) of adsorbed a) hexylphosphonic b) hexylsulfonic on $CsPbCl_3$ (110) surface with both type of cation (Pb and Cl) terminations and without surface Cl vacancies.

References

1. Protesescu, L.; Yakunin, S.; Bodnarchuk, M. I.; Krieg, F.; Caputo, R.; Hendon, C. H.; Yang, R. X.; Walsh, A.; Kovalenko, M. V., Nanocrystals of Cesium Lead Halide Perovskites ($CsPbX_3$, X = Cl, Br, and I): Novel Optoelectronic Materials Showing Bright Emission with Wide Color Gamut. *Nano letters* **2015,** *15* (6), 3692-3696.
2. Berlman, I., *Handbook of florescence spectra of aromatic molecules*. Elsevier: 2012.
3. Kandi, D.; Mansingh, S.; Behera, A.; Parida, K., Calculation of relative fluorescence quantum yield and Urbach energy of colloidal CdS QDs in various easily accessible solvents. *Journal of Luminescence* **2021,** *231*, 117792.
4. Grabolle, M.; Spieles, M.; Lesnyak, V.; Gaponik, N.; Eychmüller, A.; Resch-Genger, U., Determination of the Fluorescence Quantum Yield of Quantum Dots: Suitable Procedures and Achievable Uncertainties. *Analytical Chemistry* **2009,** *81* (15), 6285-6294.
5. Xu, K.; Vliem, J. F.; Meijerink, A., Long-Lived Dark Exciton Emission in Mn-Doped CsPbCl3 Perovskite Nanocrystals. *The Journal of Physical Chemistry C* **2019,** *123* (1), 979-984.
6. Guo, X.; Huang, Y.-T.; Lohan, H.; Ye, J.; Lin, Y.; Lim, J.; Gauriot, N.; Zelewski, S. J.; Darvill, D.; Zhu, H. J. J. o. M. C. A., Air-stable bismuth sulfobromide (BiSBr) visible-light absorbers: optoelectronic properties and potential for energy harvesting. *J. Mater. Chem. A*

2023, *11* (42), 22775-22785.
7. Wu, N.; Fu, L.; Su, M.; Aslam, M.; Wong, K. C.; Dravid, V. P. J. N. l., Interaction of fatty acid monolayers with cobalt nanoparticles. *Nano Lett.* **2004,** *4* (2), 383-386.
8. Lee, S.-Y.; Harris, M. T. J. J. o. c.; science, i., Surface modification of magnetic nanoparticles capped by oleic acids: Characterization and colloidal stability in polar solvents. *J. Colloid Interface Sci.* **2006,** *293* (2), 401-408.
9. Gao, Y.; Chen, G.; Oli, Y.; Zhang, Z.; Xue, Q., Study on tribological properties of oleic acid-modified TiO2 nanoparticle in water. *Wear*

2002, *252* (5-6), 454-458.
10. Kaewmanee, T.; Wannapop, S.; Phuruangrat, A.; Thongtem, T.; Wiranwetchayan, O.; Promnopas, W.; Sansongsiri, S.; Thongtem, S., Effect of oleic acid content on manganese-zinc ferrite properties. *Inorg. Chem. Commun.* **2019,** *103*, 87-92.
11. Hohenberg, P.; Kohn, W., Density functional theory (DFT). *Phys. Rev.* **1964,** *136* (1964), B864.
12. Kohn, W.; Sham, L. J., Self-consistent equations including exchange and correlation effects. *Phys. Rev.* **1965,** *140* (4A), A1133.
13. Perdew, J. P.; Burke, K.; Ernzerhof, M., Generalized gradient approximation made simple. *Phys. Rev. Lett.* **1996,** *77* (18), 3865.
14. Blöchl, P. E., Projector augmented-wave method. *Phys. Rev. B.* **1994,** *50* (24), 17953.
15. Kresse, G.; Furthmüller, J. J. P. r. B., Efficient iterative schemes for ab initio total-energy calculations using a plane-wave basis set. *Phys. Rev. B.* **1996,** *54* (16), 11169.

16. Kresse, G.; Furthmüller, J. J. C. m. s., Efficiency of ab-initio total energy calculations for metals and semiconductors using a plane-wave basis set. *Comput. Mater. Sci.* **1996,** *6* (1), 15-50.
17. Monkhorst, H. J.; Pack, J. D. J. P. r. B., Special points for Brillouin-zone integrations. *Phys. Rev. B.* **1976,** *13* (12), 5188.
18. Momma, K.; Izumi, F. J. J. o. a. c., VESTA 3 for three-dimensional visualization of crystal, volumetric and morphology data. *J. Appl. Cryst.* **2011,** *44* (6), 1272-1276.
19. Grimme, S.; Antony, J.; Ehrlich, S.; Krieg, H. J. T. J. o. c. p., A consistent and accurate ab initio parametrization of density functional dispersion correction (DFT-D) for the 94 elements H-Pu. *J . Chem. Phys.* **2010,** *132* (15).
20. Di Liberto, G.; Fatale, O.; Pacchioni, G. J. P. C. C. P., Role of surface termination and quantum size in α-CsPbX 3 (X= Cl, Br, I) 2D nanostructures for solar light harvesting. *Phys. Chem. Chem. Phys.*

2021, *23* (4), 3031-3040.
21. Ahmed, G. H.; El-Demellawi, J. K.; Yin, J.; Pan, J.; Velusamy, D. B.; Hedhili, M. N.; Alarousu, E.; Bakr, O. M.; Alshareef, H. N.; Mohammed, O. F., Giant Photoluminescence Enhancement in $CsPbCl_3$ Perovskite Nanocrystals by Simultaneous Dual-Surface Passivation. *ACS Energy Lett.* **2018,** *3* (10), 2301-2307.
